# Adaptive evolution by recombination is not associated with increased mutation rates in Maize streak virus

**DOI:** 10.1186/1471-2148-12-252

**Published:** 2012-12-27

**Authors:** Adérito L Monjane, Daniel Pande, Francisco Lakay, Dionne N Shepherd, Eric van der Walt, Pierre Lefeuvre, Jean-Michel Lett, Arvind Varsani, Edward P Rybicki, Darren P Martin

**Affiliations:** 1 Department of Molecular and Cell Biology, University of Cape Town, Rondebosch, Cape Town, 7701, South Africa; 2 Department of Botany and Horticulture, Maseno University, P.O. Box 333, Maseno, Kenya; 3 Kapa Biosystems, P.O. Box 12961, Mowbray, 7705, South Africa; 4 CIRAD, UMR 53 PVBMT CIRAD-Université de la Réunion, Pôle de Protection des Plantes, Saint Pierre, La Réunion, 97410, France; 5 Biomolecular Interaction Centre, University of Canterbury, Private Bag 4800, Christchurch, 8140, New Zealand; 6 School of Biological Sciences, University of Canterbury, Private Bag 4800, Christchurch, 8140, New Zealand; 7 Electron Microscope Unit, University of Cape Town, Rondebosch, Cape Town, 7701, South Africa; 8 Institute of Infectious Disease and Molecular Medicine, University of Cape Town, Observatory, Cape Town 7925, South Africa; 9 Centre for High-Performance Computing, Rosebank, Cape Town, South Africa

## Abstract

**Background:**

Single-stranded (ss) DNA viruses in the family *Geminiviridae* are proving to be very useful in real-time evolution studies. The high mutation rate of geminiviruses and other ssDNA viruses is somewhat mysterious in that their DNA genomes are replicated in host nuclei by high fidelity host polymerases. Although strand specific mutation biases observed in virus species from the geminivirus genus *Mastrevirus* indicate that the high mutation rates in viruses in this genus may be due to mutational processes that operate specifically on ssDNA, it is currently unknown whether viruses from other genera display similar strand specific mutation biases. Also, geminivirus genomes frequently recombine with one another and an alternative cause of their high mutation rates could be that the recombination process is either directly mutagenic or produces a selective environment in which the survival of mutants is favoured. To investigate whether there is an association between recombination and increased basal mutation rates or increased degrees of selection favoring the survival of mutations, we compared the mutation dynamics of the MSV-MatA and MSV-VW field isolates of Maize streak virus (MSV; *Mastrevirus*), with both a laboratory constructed MSV recombinant, and MSV recombinants closely resembling MSV-MatA. To determine whether strand specific mutation biases are a general characteristic of geminivirus evolution we compared mutation spectra arising during these MSV experiments with those arising during similar experiments involving the geminivirus Tomato yellow leaf curl virus (*Begomovirus* genus).

**Results:**

Although both the genomic distribution of mutations and the occurrence of various convergent mutations at specific genomic sites indicated that either mutation hotspots or selection for adaptive mutations might elevate observed mutation rates in MSV, we found no association between recombination and mutation rates. Importantly, when comparing the mutation spectra of MSV and TYLCV we observed similar strand specific mutation biases arising predominantly from imbalances in the complementary mutations G → T: C → A.

**Conclusions:**

While our results suggest that recombination does not strongly influence mutation rates in MSV, they indicate that high geminivirus mutation rates are at least partially attributable to increased susceptibility of all geminivirus genomes to oxidative damage while in a single stranded state.

## Background

Rapidly evolving viruses can offer unique insights into the relationships between nucleotide substitution dynamics and, for example, the evolution of multi-drug resistance [1,2], immune evasion [3], vaccine escape [4], host range variation [5,6] and host resistance-breaking [7-10]. Much of the adaptive potential of viruses stems from their large population sizes and, in many instances, their high degrees of genetic variability. In the case of RNA viruses the use of error-prone RNA polymerases during replication is the primary driver of high basal mutation rates [11] – defined here as the basal rate at which mutations arise. Many RNA viruses also have high substitution rates – defined here as the rate at which mutations become established within populations – which reflects the probability of arising mutations becoming fixed within virus populations, and is subject to the effects of processes such as natural selection [12,13], competition with other mutations [14], and overall viral population dynamics [15,16].

However, among DNA-containing viruses, which typically have much lower mutation rates than RNA viruses, the substitution rates of many single-stranded (ss) DNA viruses have been estimated to be the in order of 10^-3^ to 10^-5^ substitution per site per year [5,17-19]: substitution rates approaching those typical of RNA viruses [20,21]. Since ssDNA viruses replicate using their hosts’ high fidelity DNA polymerases [22], additional mutagenic factors have been sought to explain their paradoxically high mutation rates.

Such factors might include chemical or enzyme-induced methylation and deamination of nucleotide bases [23,24], genomic secondary structure-induced replication errors [25], or the mode of virus replication (with the stamping machinery mode of replication expected to result in lower mutation rates than geometric replication [26,27]).

The ssDNA viruses of the family *Geminiviridae* display substantial mutation biases that could potentially indicate the cause(s) of their high mutation rates. In both field isolated geminiviruses in the genus *Begomovirus*[28] and mutants arising during evolution experiments involving geminiviruses in the genus *Mastrevirus*[18,29], particular oxidative-damage related mutations such as G → T, C → T and G → A apparently occur more frequently than other mutations*.* In the mastreviruses one of these mutations, G → T, occurs in a markedly strand-specific manner [29]. It is however, currently unknown whether or not such strand-specific mutation biases are a general feature of geminivirus evolution.

Another factor potentially influencing both the substitution and mutation rates of geminiviruses is genetic recombination, which in some instances has been shown to be mutagenic [30-32]. The possible interplay between mutation, substitution and recombination rates is particularly interesting when considering the geminiviruses. Both rampant recombination and high nucleotide substitution rates [33] are credited with the emergence of many of the geminivirus species that today threaten the production of various important food crops [34,35]. Among the most important of these species is *Maize streak virus* (MSV), variants of which seriously constrain maize production throughout sub-Saharan Africa [36].

MSV has a single component, circular, ~2.7 Kb ssDNA genome that encodes only four proteins: Replication-associated proteins Rep and RepA in the complementary-sense [37-41], and a movement protein (MP) and coat protein (CP) in the virion-sense [37,42-44]. A long intergenic region (LIR) [45,46] separates the start codons of the complementary and virion-sense genes, which in turn converge on a short intergenic region (SIR).

To determine first, whether an association exists between mutation and recombination events in MSV and second, whether strand specific mutation biases are a general feature of geminivirus evolution, we analysed in controlled laboratory infections, the mutation dynamics of field isolated MSV genomes relative to those of (1) a laboratory constructed MSV recombinant, (2) MSV recombinants that emerged during a previously reported MSV evolution experiment [47], and (3) Tomato yellow leaf curl virus (TYLCV) mutants that emerged during another previously reported evolution experiment [48]. Specifically, we intended to answer the following questions: (1) Is the process of MSV recombination mechanistically mutagenic, (2) is there an adaptive period following recombination during which nucleotide substitution rates are elevated and, (3) are the mutation spectra arising during controlled MSV and TYLCV evolution experiments indicative of conserved strand-specific mutation biases.

## Methods

### Viruses

Agroinfectious clones of the wild-type (wt) MSV isolates, MSV-VW [49] and MSV-MatA [50], have been described previously. However, MSV-VW and MSV-MatA-derived mutant genomes arising during evolution experiments are introduced here for the first time (see Additional files 1 and 2). Similarly, mutants of the laboratory constructed recombinant genome, VWMPCPLIRMat [51] are also mentioned here for the first time, whereas mutated, mostly MSV-MatA-like, recombinant genomes (see Additional files 3, 4 and 5), have been reported previously [47] and are re-analysed here. TYLCV derived mutant genomes arising during evolution experiments conducted by Martin *et al*. [48] are described here for the first time.

### Agro-infection of maize

We agroinoculated five three-day-old seedlings of the MSV-sensitive maize genotype Sweetcorn cv. Golden Bantam (GB; Millington Seed Co. USA) with MSV-MatA or MSV-VW (five plants per virus) as described previously [52]. At 101 days post inoculation (dpi) we isolated viral DNA from a single symptomatic leaf of each of the ten infected plants. Similarly, we inoculated 18 GB seedlings using the VWMPCPLIRMat chimaera and isolated viral DNA from each plant between 55 and 68 (average of 61 days) dpi.

### Viral DNA isolation, cloning and sequencing

Viral DNA was isolated using the Extract-n-Amp™ kit (Sigma-Aldrich), followed by rolling-circle amplification as previously described [53,54]. Amplified DNA was digested with the restriction enzyme *Bam*HI to generate ~ 2.7 kb monomeric MSV genomes which were gel-purified (GFX™, GE Healthcare), ligated to *Bam*HI-digested pGEM^®^-3Zf(+) (Promega Biotech) using T4 DNA ligase (Fermentas), and used to transform competent *Escherichia coli* (*E*. *cloni*^®^, Lucigen^®^ Corporation) using standard protocols [55]. From each plant we randomly selected between one and six cloned genomes for sequencing (Macrogen, Korea) using universal M13 forward and reverse sequencing primers and previously-described MSV-specific primers [56].

### Analysis of sequence variability

Within all of the different viral genomes used to initiate the various evolution experiments we calculated the expected frequencies of each of the twelve possible nucleotide mutations (i.e. all of the possible transitions and transversions), as outlined previously [29]. Similarly, deviation of observed mutation frequencies from their expected frequencies was detected using a two-tailed chi-square test as in van der Walt *et al*. [29] which explicitly accounts for differences in parental virus nucleotide compositions. The observed and expected number of transition (Ts) and transversion (Tv) mutations were calculated by summing up the frequencies of all observed and expected Ts and Tv mutations, respectively. The observed Ts and Tv mutation frequencies were collectively tested for significant deviation from random expectations using the same chi-square test.

We expressed the mutation rate of each of the different viruses used to initiate the various evolution experiments as the number of mutations occurring per nucleotide, per year (muts/site/year). For each individual plant, we first divided the number of mutations observed in the viral progeny by the number of nucleotides sequenced (that is, from between one and six viral genomes), then divided this number by the time in years between inoculation and when the genomes were sampled to obtain the mutation rate per plant. We then calculated the mutation rates of each of the viruses used to initiate the evolution experiments as the average mutation rate across all of the plants inoculated with these viruses.

### Detection of selection across entire genome regions

To calculate the significance of mutations in our evolutionary experiments occurring in sites deemed to be evolving under negative or non-negative (i.e. both positive and neutral) selection pressure in the MSV-A genome, we used the observed frequency of mutations under the categorical variables, type of selection and type of mutation, to calculate a two-tail p-value (Fisher’s Exact Test) using a 2X2 contingency table. Whereas we hypothesised that genes evolving under positive selection might accumulate more non-synonymous mutations than expected, we hypothesised that those evolving under negative selection might accumulate fewer non-synonymous mutations than expected.

We used a two-tailed Fisher’s exact test to determine whether mutations occurred more or less frequently in coding regions than in non-coding regions, by comparing the numbers of mutations observed over these respective regions in VWMPCPLIRMat-derived genomes and recombinant MSV genomes (2,219 nts, and 470 nt in each region, respectively), MSV-VW-derived genomes (2211 nt and 471 nt, respectively), and MSV-MatA-derived genomes (2226 nt and 463 nt, respectively).

To further infer the types of selection pressure acting on individual genes of VWMPCPLIRMat, MSV-VW and MSV-MatA, we used the random effects likelihood (REL), fixed effects likelihood (FEL), internal fixed effects likelihood (IFEL), and single-likelihood ancestor counting (SLAC) methods implemented in the web-server Datamonkey (http:http://www.datamonkey.org) [57,58]. For this analysis, we used the F81 [59] nucleotide substitution model determined to be the best fit model for each dataset analysed (see Additional file 6). We also conducted almost identical analyses on the coding regions of publically available MSV-A full genome sequences (358 in March 2011). The only difference in the analyses of the publically available sequences was the use of the generalized time reversible (GTR) nucleotide substitution model which was determined to be the best fit model using the computer program RDP3 [60] and the inference of codon specific synonymous:nonsynonymous substitution ratios.

## Results and discussion

In order to determine first, whether in MSV genomes the recombination process is itself mutagenic (and therefore causes an increase in the basal mutation rate) and second, whether recombination generates a selective environment where there is an increased frequency of adaptive mutation (and therefore an increased substitution rate) [61,62], we infected maize plants with the wild-type (wt) viruses MSV-MatA (a maize-adapted virus) and MSV-VW (a grass-adapted virus), and a laboratory-constructed chimaera of these, VWMPCPLIRMat. We allowed these viruses to evolve within the infected plants for between approximately 60 and 101 days and then conducted an analysis on the mutations generated within that time. Similarly, we analysed the mutation dynamics of the recombinant MSV genomes (some of which had evidence of up to 22 recombination breakpoints) isolated from infected maize plants by Monjane *et al*. [47].

Collectively, we sequenced and analysed 27 MSV-MatA derived, 16 MSV-VW derived and 18 VWMPCPLIRMat derived genomes. We additionally analysed 49 recombinant MSV genomes previously isolated from MSV-sensitive plants [47], and 142 TYLCV derived genomes described by Martin *et al.*[48].

### The distribution of mutations arising within the MSV genome

**Figure 1:**
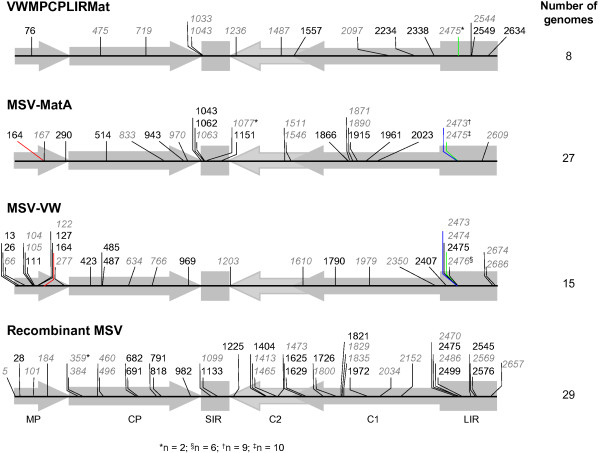
**Distribution of mutations in recombinant and wild**-**type MSV genomes. **Under the name of each group of mutants (that is, VWMPCPLIRMat, MSV-MatA, MSV-VW, and the VWMPCPMat + MatMPCPVW and VWMPCPLIRMat + MatVMPCPLIRVW-derived recombinant MSV) the distribution of mutations is a composite of all the unique mutations identified in each population of viruses. Whereas the numbers that are written in italics and coloured in grey indicate sites where transversion mutations were detected, those in regular font and in black colour indicate sites where transition mutations were detected. These numbers are connected by vertical lines which refer to the mutated nucleotide of a specific virus listed in Additional file 7, and for simplicity we use the genomic co-ordinates of MSV-MatA to pinpoint the genomic position of each mutation. The lines are predominately drawn in black. However, those lines in red, blue or green indicate genomic positions (among at least a pair of viruses belonging to the different virus population groups) where identical mutations were detected. Those genomic sites where at least two viruses isolated from different plants have a particular convergent mutation are indicated using the symbols *, †, ‡ and §. At the bottom of the panels, MP (movement protein), CP (coat protein), SIR (short intergenic region), C2 + C1 (replication-associated proteins), and LIR (long intergenic region) delineate MSV open-reading frames and intergenic regions.

To visualize the distribution of unique mutations (i.e. mutations were counted only once irrespective of how many plants they were observed in), we mapped the sites at which they occurred (Figure 1). Of the 104 unique mutations identified among all the analysed genomes (i.e. 14, 23, 24, and 36 unique mutations identified in plants inoculated with, respectively, VWMPCPLIRMat, MSV-MatA, MSV-VW, and recombinant MSV genomes), seven mutations occurred independently in multiple different plants (see Additional file 7).

In all the virus populations analyzed, mutations were observed in all the genomic regions (Figure 1). However, while there was no statistically significant difference between the numbers of mutations observed in the coding and non-coding regions of viruses derived using VWMPCPLIRMat, MSV-VW or the recombinant MSV genomes (p = 0.179, 0.082, 0.202, respectively; Fisher’s exact test), there were disproportionally more mutations within the non-coding regions of MSV-MatA mutants (p = 0.008; Fisher’s exact test). This concentration of mutations in the non-coding regions of maize-adapted MSV mirrors that seen in *East African cassava mosaic Cameroon virus* (EACMCV; a geminivirus in the genus *Begomovirus*) propagated in cassava [62]. Relatively fewer mutations within the coding regions of MSV-MatA than in the other viruses studied may reflect the fact that MSV-MatA proteins are already highly maize adapted whereas those in MSV-VW, the VWMPCPLIRMat chimaera and the various recombinants are not as well adapted and have therefore experienced higher rates of nucleotide substitution due to the fixation of adaptive mutations over the course of our experiment. In addition to host adaptation in VWMPCPLIRMat and the recombinant viruses, it is possible that increased mutation rates in coding regions may also be associated with adaptation of genes derived from different parental viruses to one another.

### Convergent mutations may or may not be adaptive

Although we generally assume here that the basal MSV mutation rate is constant across the genome, it should be pointed out that there are instances where virus mutation rates have been found to vary greatly from site to site within a genome [63]. Here we use our mutation data to establish whether regions prone to mutation might exist within the MSV genome.

Of the 104 unique mutations identified, 97 were only ever seen in viruses originating in individual plants. Seven, however, occurred at sites that may be hot-spots for mutations in MSV because: (1) among these, convergent mutations occurred at some sites in between two and six independently-infected plants (for example, the G(359)T mutation detected in a pair of recombinant viruses, the G(164)A mutation in both a MSV-MatA derived virus and a MSV-VW-derived virus, the T(2473)G mutation seen in nine MSV-MatA and one MSV-VW derived viruses (viruses isolated from five plants independently infected with MSV-MatA, and one plant infected with MSV-VW; All genome coordinates are relative to the MSV-MatA genome; see Additional file 7); (2) two individual sites experienced two or three divergent mutations (for example, the A(2475)G/T/C mutations in MSV-MatA, MSV-VW, recombinant MSV, and VWMPCPLIRMat derived viruses and the T(2476)C/G mutation in MSV-VW derived viruses); and (3) clusters of mutations were observed at adjacent sites, for example 22 independent mutation events were collectively detected across the various experiments at positions 2473, 2474, 2475 and 2476; see Additional file 7). Whereas three of the potential mutation hot-spots were found within the LIR (two unique convergent mutations were detected at two of these sites accounting for four of the five detected convergent mutations in the LIR), one each was found within the *mp* and *cp* genes.

It is important to point out here that recurrent convergent mutations at individual genome sites have been reported previously in geminivirus evolution experiments. In the case of MSV, a nucleotide site within the portion of the *rep* gene encoding the retinoblastoma-related protein binding domain was observed to rapidly and repeatedly mutate from an atypical MSV-A polymorphism to one that is conserved in all known field isolated MSV genomes [64,65]. Similarly, a readily mutable nucleotide site has been reported in plants independently-infected with the begomoviruses Tomato yellow leaf curl China virus (TYLCCV [66]), and Tomato golden mosaic virus (TGMV [67]). In all of these studies, recurrent convergent mutations were likely adaptive, in that they probably restored functional nucleic acid secondary structures and/or protein interaction motifs.

Due to their compact, structurally simple, and yet functionally complex genomes, ssDNA viruses such as MSV would be expected to have a low degree of mutation robustness [14,68], such that the vast majority of arising mutations would be expected to be at least slightly deleterious. One would therefore expect the basal mutation rate to be substantially higher than the substitution rate. With this in mind our observation of seven convergent mutations in different independent infections indicates that either the observed mutations are neutral (or perhaps only mildly deleterious) but occur at sites where the mutation rate is much higher than elsewhere in the genome, or that the mutation rate is uniformly high throughout the MSV genome and the mutations at these sites are selectively favoured because they are beneficial. We therefore investigated the potential biological implications of these convergent mutations.

The G(359)T mutation observed in a pair of recombinant MSV viruses (see Additional file 7) occurred within the potential nuclear localization signal domain located within the *cp* of MSV [69], at a site where purine bases are conserved in all known MSV strains (data not shown). This mutation caused a non-synonymous amino acid change (see Additional file 7) from a serine residue with a short hydrophilic side-chain at position 15 of the CP to an isoleucine residue with a large hydrophobic side-chain: a change that would be expected to substantially alter the N-terminal structure of CP relative to that of all other known MSV isolates. It is therefore unlikely that this mutation was completely neutral.

A G(164)A mutation in the *mp* of one MSV-MatA derived virus and one MSV-VW-derived virus occurred at a site encoding a residue within the hydrophobic potential trans-membrane domain of MP [70]. Amongst almost all known field isolated MSV genomes (there is only one exceptional genome out of 581; data not shown), a guanine base is conserved at this site. This mutation would have resulted in a relatively conservative amino acid change from a hydrophobic valine residue to a hydrophobic isoleucine residue and it is therefore possible that the mutation was selectively neutral.

Intriguingly all five of the convergent mutations in the LIR and two additional unique mutations, were associated with the TATA box positioned 101 nt upstream of the *rep* start codon. Whereas the convergent T(2473)G mutations were observed in MSV-MatA and MSV-VW derived genomes, the convergent A(2475)G mutations were observed in recombinant MSV, MSV-MatA, MSV-VW, and VWMPCPLIRMat derived genomes, the A(2475)T mutation was observed in MSV-MatA and VWMPCPLIRMat derived genomes, and both the T(2476)C/G mutations were observed only in MSV-VW derived genomes (see Additional file 7 and Figure 1).

It is perhaps because base changes within the TATA boxes of promoter elements will adversely affect transcription [71,72] that only a very small number of wt MSV isolates (data not shown) have polymorphisms within the above-mentioned TATA box. The high degree of sequence conservation observed between nucleotide positions 2473 and 2475 in wt MSV isolates strongly suggests that the mutations observed at these sites in our experiment are probably deleterious.

In fact, the deleterious effects of a point mutation within this TATA box has already been experimentally verified in the wt MSV-A isolates, MSV-Ns (GenBank accession number: X01633) and MSV-Nm. Although these isolates differ by only three nucleotides, the former induces considerably more severe symptoms in maize plants [71]. However, a A(2473)G mutation in MSV-Ns (a site that is homologous to that at which we detect the A(2475)G/T/C mutations) and the converse G(2473)A mutation in MSV-Nm had a substantial influence on symptom severity in maize [71]. The fact that the G to A mutation in the MSV TATA box enhanced the virulence of MSV-Nm, and the reverse mutation (i.e. A to G) significantly decreased the virulence of hybrids of these genomes [73], supports the hypothesis that the A(2475)G/T/C and other TATA box mutations we observe are likely to be at least mildly deleterious. One cannot exclude the possibility, however, that virulence does not reflect virus fitness in this case. For example, mutations that reduce the virulence of MSV variants might, within the context of a mixed infection with wild-type highly virulent variants (i.e. situations where some degree of genetic complementation is likely), increase the replicative capacity of these mutants relative to their more virulent progenitors.

Of the seven convergent mutations that we observed it seems unlikely that either the *cp* mutation or the TATA box mutations are neutral. If these non-neutral mutations are deleterious, it would indicate that these particular genome sites must have extremely high mutation rates (especially those in the TATA box) to account for the fact that they have been observed despite selection acting to remove them. It is perhaps therefore more plausible that these mutations are at least slightly adaptive within the context of the specific infections where they arise, and that their observation in multiple plants reflects the fact that whenever these mutations arise within an MSV infection of the maize genotype GB, they are selectively favoured to the point where they attain frequencies high enough to be detectable. The absence of analogous mutations in field isolated MSVs may reflect the fact that these mutations are possibly only adaptive within the specific infections where they arise because these infections also contain wild-type genomes capable of complementing the mutants. Following transmission the mutant viruses might not be able to survive in the long-term due to the fact that they will eventually become separated from co-infecting viruses that do not carry the mutations.

### Evidence of selection acting on mutant genomes

Of the various virus genotypes analysed here, MSV-MatA is the most maize-adapted followed by the recombinant MSV genomes, VWMPCPLIRMat and MSV-VW [47]. Given their particularly reduced fitness in maize we expected that during the course of our evolution experiments VWMPCPLIRMat and MSV-VW-derived viruses might display evidence of increased rates of adaptive evolution. Whereas in the case of MSV-VW and the MSV-VW derived portions of VWMPCPLIRMat (i.e. the SIR, *rep* and ~ 85 nts of the LIR) these adaptive mutations could have been those that optimised virus-host molecular interactions [46,51], in the case of VWMPCPLIRMat these could additionally have included mutations that re-established vital sequence specific interactions between the MSV-MatA and MSV-VW derived portions of its genome [51,74]. Specifically, we expected that the accumulation of adaptive mutations in VWMPCPLIRMat and MSV-VW derived viruses might be evident from the spectra of the mutations identified within these viruses.

Firstly though, to infer the specific types of selection pressures acting on field isolated variants of the maize-adapted MSV-A strain, we calculated the ratio of the rates of non-synonymous (*d*_*N*_) and synonymous (*d*_*S*_) mutations (i.e. the *d*_N_/*d*_S_ ratio) arising within coding regions using the codon-based maximum likelihood-based selection detection methods REL, IFEL, FEL, and SLAC implemented in the web-server Datamonkey [57,58]. We identified every codon within the MSV-A genome with some evidence (an associated p-value <0.05) of evolution under either positive or negative selection pressures (see Additional file 8). We then assessed whether the unique mutations identified in the coding regions of our various evolution experiments occurred in the codons inferred to be evolving non-neutrally within the genomes of field isolated MSV-A isolates.

Using a Fisher’s exact test, we were unable to find any significant associations in any of the experiments between the locations of observed synonymous and non-synonymous mutations and sites within the MSV-A genome respectively evolving in nature under either negative or positive selection. We were also unable to detect any such associations when we combined the data sets (see Additional file 9), suggesting that the 78/104 mutations that were observed to occur within coding regions during our evolution experiments are not obviously subject to strong selection pressures in the field.

In three of the four experimental mutant data sets (specifically excluding the recombinant virus data set because recombination invalidates *d*_N_/*d*_S_ based selection analyses in this data set) the mean *d*_N_/*d*_S_ values of almost all of the protein coding genes were < 1, suggesting pervasive negative selection (i.e. selection against non-synonymous base changes; see Additional file 6). Exceptionally, however, in the MSV-VW experiment the *cp* was inferred to be evolving under weak positive selection (mean *d*_N_/*d*_S_ = 1.92 and 1.90 using the SLAC and REL methods, respectively), and, while the MSV-VW *mp* was on average inferred to be evolving under weak negative selection (mean *d*_N_/*d*_S_ = 0.64 and 0.68 using the SLAC and REL methods, respectively), it contained two codons (p-value < 0.05) encoding amino acids within the MP hydrophobic domain (a probable trans-membrane domain [70]) that were inferred to be evolving under positive selection using the REL method (see Additional file 6).

Although one should be cautious when it comes to interpreting these observations - due to the low numbers of mutations observed in the experimental viruses relative to those enumerated during the analyses of the field isolates - it is nonetheless interesting that the codons in the *mp* inferred to be evolving under positive selection in the MSV-VW-derived viruses are apparently evolving under predominantly neutral selection in the field isolated MSV-A sequences (see Additional files 6 and 8). Also, because MSV-VW is the least maize-adapted virus we analysed, it is the virus that we most expected to display some evidence of evolution under positive selection in maize. The fact that the weak signals of positive selection detected within MSV-VW are specific to the *mpcp* gene module is also potentially significant. This genome region is both the main pathogenicity determinant in maize of the maize adapted MSV-A strain [51] and its transfer by recombination from a *Digitaria* adapted MSV-B-like virus to a *Digitaria* adapted MSV-G or –F-like virus is credited with the genesis of the progenitor of all MSV-A isolates [75].

### Recombinant and wild-type MSV genomes display similar mutation rates

Besides the probability that recombinant viruses might experience elevated degrees of positive selection favouring the restoration of recombinationally disrupted intra-genome interactions (a process that might be associated with increased numbers of detected mutation events), it is also plausible that the recombination process itself might be inherently mutagenic [31,32]. We therefore compared the mutation rates of the laboratory constructed recombinant, VWMPCPLIRMat, and recombinant MSV genomes [47], with those of the wt MSV-MatA and MSV-VW genomes.

Within the recombinant MSV genomes we deduced mutation rates of 1.77 × 10^-3^ muts/site/year based on a total of 39 mutations detected in 131,761 sequenced nts from 49 recombinant genomes (see Additional file 10). For VWMPCPLIRMat a mutation rate of 1.99 × 10^-3^ muts/site/year was calculated based on 16 mutations detected in 48,402 sequenced nts from 18 genomes. Both of these rates were slightly lower (albeit not significantly so) than those determined for MSV-MatA (2.02 × 10^-3^ muts/site/year determined from 41 mutations detected in 72,603 sequenced nt from 27 genomes; see Additional file 10) and MSV-VW (2.41 × 10^-3^ muts/site/year determined from 33 mutations detected in 42,912 sequenced nt from 16 genomes; see Additional file 10).

The fact that the estimated mutation rate of the MSV-A-like recombinants arising during recombination experiments was lower than that of all the other examined groups of genomes strongly suggests that the mechanistic process of recombination in MSV is probably not mutagenic. Similarly, the fact that the laboratory constructed recombinant genome, VWMPCPLIRMat accumulated mutations at a lower rate than both the MSV-MatA and MSV-VW genomes indicated that it was probably not subject to higher degrees of positive selection acting on arising mutants than non-recombinant genomes.

It is important to note, however, that our wild-type and recombinant genomes were not all sampled at exactly the same times post infection. The wild-type genomes were, in fact sampled on average 41 days later than the recombinants and were, relative to the recombinants, subject to selective pressures for longer periods. The overall effect of this increased selection should have been to reduce, at least slightly, the mutation rates observed in the wild-type genomes relative to those in the recombinant genomes [68]. Nevertheless, the fact that the mutation rates observed in the wild type genomes were actually higher than those observed in the recombinant genomes therefore even more strongly supports the assertion that recombination is not associated with elevated mutation rates.

Considering the lack of any evidence of elevated substitution rates that may have been driven by higher degrees of selection acting on the recombinant genomes, it is important to note that we nevertheless detected one very clear signal of selection within the distribution of mutations that we identified. Specifically, the non-coding regions of viruses in all of the MSV experiments accumulated mutations at over twice the frequency of the coding regions: 4.34 × 10^-4^ mut/site versus 2.67 × 10^-4^ mut/site for the recombinant MSV genomes, 5.91 × 10^-4^ mut/site/year versus 2.75 × 10^-4^ mut/site for VWMPCPLIRMat, 14.8 × 10^-4^ mut/site versus 3.7 × 10^-4^ for MSV-MatA, and 11.3 × 10^-4^ mut/site versus 5.6 × 10^-4^ mut/site for MSV-VW (see Additional file 10). Among geminiviruses such a trend is apparently not unique to MSV [17,62,66,76,77], and this is likely attributable to generally reduced negative selection pressures acting on geminivirus non-coding regions [28].

It should also be stressed here that it is very likely that the mutation spectra observed in our short-term evolution experiments were considerably less influenced by selection than those encountered in either longer-term evolution experiments, or viruses sampled from nature. The MSV genome-wide mutation rates determined in our short-term evolution experiments (i.e. experiments lasting less than four months) are 8–12 times higher than those determined in longer term experiments spanning one to 32 years (~0.2 × 10^-3^ – 1.05 × 10^-3^ subs/site/year,[18,29,78], but are very close to mutation rates determined in evolution experiments carried out over a similar time-frame for the geminiviruses TYLCCV (between 2.49 and 1.61 × 10^-3^ subs/site/year [66]; and TYLCV (1.4 × 10^-3^ subs/site/year [79]). These differences in mutation rates between short- and long-term evolution experiments are entirely consistent with the hypothesis that in short-term experiments negative selection has less of a modulating influence on mutation frequencies than in longer term experiments. Therefore, the mutation rates calculated in our short-term evolution experiments may reflect a closer approximation to the basal MSV mutation rate than previously determined from longer-term experiments which are likely to have been more strongly influenced by selection and genetic drift [68,80-82].

It is noteworthy that these short-term mutation rates approximate the substitution rates typical of fast-evolving RNA viruses [20,21]. To explain the high mutation rates of geminiviruses, it is thought likely that replicating geminivirus DNA remains un-methylated [83] and as a consequence of this it is unaffected by the corrective mismatch repair systems of the host cells upon which they rely for replication [84]. Similarly, the base-excision repair mechanisms of host cells may also be ineffectual because geminivirus DNA is only transiently double-stranded during rolling-circle replication [28]. Furthermore, because geminiviruses spend a significant amount of time in a single-stranded state, high mutation rates may also be explained by spontaneous or enzyme-induced deamination of unpaired DNA bases [85-88]. In MSV, the effects of a predominantly single-stranded existence is perhaps manifest in strand specific nucleotide mutation biases that are indicative of spontaneous mutations occurring predominantly on the virion-strand [18,29].

### Strand-specific mutation biases in geminiviruses

To further explore the possibility that virion-strand specific mutation biases are a feature of geminivirus evolution in general, we determined with greater precision than has been previously achieved with much smaller data sets the nucleotide mutation spectra of MSV (a mastrevrius), and TYLCV (a begomovirus). Our TYLCV data set was derived from a set of sequences obtained during evolution experiments described by Martin *et al*. [48] that were carried out over a comparable time-frame (120 days) to those described here. It was unfortunately not possible to pool our data with nucleotide mutation frequency data obtained in various other published evolution experiments, where either input virus sequences were unknown and mutation counts were inferred by maximum likelihood or parsimony based approaches [17,18,28], or where observed mutations were derived only from small portions of the virus genome [66].

Using the number of mutations observed in our MSV and TYLCV data sets, we assumed that a bias towards mutations occurring on the virion-strand would be evident in the disproportionate numbers of complementary mutations: for example, A → G mutations are complementary with T → C mutations and are expected to occur at almost exactly the same frequency if there are no strand-specific mutation biases. Assuming that complementary mutations are approximately equally probable (our null hypothesis) we calculated the binomial and two-tailed binomial probabilities associated with the A → G mutation rate equalling the T → C mutation rate, the A → C mutation rate equalling the T → G mutation rate, the G → A mutation rate equalling the C → T mutation rate, and the G → T mutation rate equalling the C → A mutation rate. The reversible mutations, A → T and T → A, C → G and G → C were excluded because these can yield no information on potential strand-specific mutation biases.

For the recombinant MSV data we detected the same imbalance between G → T : C → A mutations (8:2 mutations; p = 0.132) as has been detected previously in long-term evolution experiments involving MSV [18,29], and the mastrevirus *Sugarcane streak Reunion virus* (SSRV [18]; Table 1). Importantly, when the mutations from the recombinant MSV data, VWMPCPLIRMat and wt isolates (MSV-MatA and MSV-VW) were combined with those obtained previously in evolution experiments involving the field isolates MSV-Tas, MSV-Set and MSV-Kom and MSV-Kom/Set chimaeric viruses [29], further imbalances in G → T : C → A (29:9 mutations; p = 0.004), G → A : C → T (18:30 mutations; p = 0.052), and A → G : T → C (16:6 mutations; p = 0.054) were detected. In the TYLCV data set, however, we detected imbalances only between G → T : C → A (46:30 mutations; p = 0.051) and A → G : T → C (8:22 mutations; p = 0.02) mutations (Table 1). These results suggest that strand-specific mutation biases that are indicated by excessive G → T mutations occurring on the virion-strand (p-value < 0.051 in the case of both MSV and TYLCV) are probably conserved throughout the geminiviruses, and other biased mutations such as excessive G → A, A → G and C → T mutations may be particular to certain species or genera.

**Table 1:** **Step**-**down multiple testing corrected binomial probability support for strand**-**specific nucleotide mutation biases in MSV and TYLCV** (**Two**-**tailed probabilities in brackets**)

**Mutation**	**Recombinant data combined**^**a**^	**VWMPCPLIRMat**	**MSV**:**MatA**	**MSV**:**VW**	**MSV data combined**^**b**^	**MSV data combined**^**c**^	**TYLCV**^**d**^
A → G : T → C	2 : 2	2 : 1	4 : 0	1 : 1	9 : 4	16 : 6	8 : 22
Binomial probability	0.750 (1.000)	1.000* (1.000*)	0.252 (0.500)	0.500 (1.000*)	0.174 (0.801)	0.054 (0.157)	0.020 (0.064)
A → C : T → G	2 : 0	1 : 1	3 : 1	0 : 5	6 : 6	11 : 10	10 : 12
Binomial probability	0.250 (1.000)	1.000 (1.000*)	0.500 (1.000*)	0.124 (0.252)	0.904 (1.000)	0.168 (1.000)	0.154 (0.832)
G → T : C → A	8 : 2	3 : 1	1 : 2	1 : 2	13 : 7	29 : 9	46 : 30
Binomial probability	0.132 (0.436)	1.000 (1.000*)	0.375 (1.000)	0.760 (1.000*)	0.222 (0.526)	0.004 (0.007)	0.051 (0.254)
G → A : C → T	4 : 11	1 : 3	3 : 6	5 : 6	13 : 26	18 : 30	48 : 55
Binomial probability	0.168 (0.354)	1.000 (1.000*)	0.492 (1.000*)	0.675 (1.000*)	0.060 (0.212)	0.052 (0.222)	0.124 (1.108)

^a^Recombinant genomes obtained using the MatMPCPVW + VWMPCPMat and MatMPCPLIRVW + VWMPCPLIRMat parental chimaeric MSV genomes [47].

^b^Data combined using values from this study only.

^c^Data combined using values from this study and from van der Walt *et al.*[29].

^d^Data obtained from Martin *et al*. [48]*The calculated step-down multiple tested corrected p-values greater than 1 are simply represented as having a p-value of 1.

### Other features of the mutation spectra that are conserved among geminiviruses

While significant differences in the frequencies of complementary mutations are indicative of general strand-specific mutation biases, we noted, as have others, that particular mutations were likely over-represented amongst the spectra of mutations that we observed in our various evolution experiments.

For example, while there was no statistical support for the number of transition (Ts) or transversion (Tv) mutations being either over- or under-represented than would be expected in most of our experiments, there was evidence of an over-representation of Ts mutations (p = 0.046) among the recombinant MSV genomes obtained using the MatMPCPLIRVW + VWMPCPLIRMat parental viruses (see Additional file 10). Moreover, when the data from the individual experiments were combined we detected an over-representation of Ts mutations (p = 0.011; see Additional file 10). Similarly, an over-representation of Ts mutations (p = 0.0002), coupled with a statistically supported under-representation of Tv mutations (p = 0.01; see Additional file 10), was detected in the TYLCV data set of Martin *et al*. [48].

In good agreement with other studies on mutation biases in mastreviruses [18,29], with the exception of the VWMPCPLIRMat data set (p = 0.12), we observed significantly higher than expected rates of C → T mutations in all of our other MSV data sets (p–values consistently lower than 0.012) and in the TYLCV data set (p < 1.0 × 10^-8^; see Additional file 10).

Among the recombinant MSV genomes obtained using the MatMPCPVW + VWMPCPMat parental viruses, and in the TYLCV data set, we also observed a significantly higher than expected rate of G → T mutations (p = 0.002 and p = 5.2 × 10^-7^, respectively). However, contrary to previous studies on the mutation dynamics of MSV, where more frequent than expected G → A mutations, as well as lower than expected C → A, T → A and T → G mutations [18] were detected, neither in our individual data sets nor combined data set (see Additional file 10 and Additional file 11) were the increased or decreased frequencies of these mutations statistically supported (see Additional file 10 and Additional file 11). However, once the data were combined there was an overall lower than expected G → C mutation frequency (p = 0.040; see Additional file 10).

Likely due to the large numbers of mutations considered in the TYLCV data set, and the fact that Ts mutations were observed to occur at a significantly higher rate than Tv mutations in this virus, six of the 12 possible nucleotide changes (five transitions and one transversion) were significantly less frequent than expected by chance, whereas four (two transitions and two transversions) were significantly more frequent (all p-values < 0.05; see Additional file 10). Crucially, in all cases where mutations were significantly over-represented in any one of the various MSV data sets (or under-represented when the data sets were combined) they were accordingly over- or under-represented in the TYLCV data set. As has been pointed out previously [28,29], these mutations (specifically, the C → T, G → A and G → T mutations) are consistent with the notion that, relative to double-stranded DNA, ssDNA may experience elevated degrees of ssDNA targeted oxidative or enzyme-induced deamination reactions [85,88,89].

## Conclusions

We have analysed the mutation dynamics of wt MSV isolates (MSV-MatA and MSV-VW), a laboratory-constructed chimaeric MSV and various recombinant viruses emerging during recombination experiments, and compared MSV mutation spectra with those occurring within the related begomovirus species, TYLCV.

Whereas we find that neither recombination nor overall degrees of host adaptation in our short-term evolution experiments are associated with obvious overall variations in mutation frequencies, we cannot exclude the possibility that either recombination between different sets of parental geminivirus genomes, or selection of recombinants over multiple transmission cycles between a variety of different hosts in the field might have an influence on longer-term geminivirus substitution rates.

Nevertheless even within our short-term experiments we find consistently elevated mutation rates within the non-coding genome regions. Our detection of pervasive negative selection within the coding regions of both our experimental recombinants and field isolated viruses is in keeping with the notion that these differences are due to negative selection acting less strongly on non-coding regions than it does on coding regions. Despite evidence of sporadic instances of possible positive selection (convergent mutations and high ratios of non-synonymous to synonymous mutations in certain codons), the fact that we detect vastly more evidence of negative selection than positive selection within the coding regions of MSV indicates that the very high MSV nucleotide mutation frequencies that we have reported here are probably still substantially lower than the virus’ basal mutation rate.

Finally, we capitalise on the high numbers of mutations identified both in our MSV experiments and in a related study for the begomovirus, TYLCV [48], to firstly confirm that previously detected strand-specific nucleotide mutation biases in MSV are a general phenomenon during geminivirus evolution and, secondly, that there exist consistent trends within the mutation spectra of all geminiviruses that are likely indicative of common mutational stresses acting on their genomes whilst they are in a single stranded state.

## Competing interests

The authors declare that they have no competing interests.

## Authors’ contributions

ALM, DP, EvdW, DPM, EPR conceived the study and participated in its design. ALM, FL, DPM, PL isolated, cloned and sequenced the viral genomes and performed basic viral bioinformatics. ALM, DPM, FL, DP analyzed and interpreted the data. ALM, DPM, DNS, EvdW, AV, EPR, DP, FL drafted and revised the manuscript. All authors read and approved the final manuscript.

## Supplementary Material

Additional file 1 **Alignment of MSV-VW-derived mutant genomes. **Full-genome sequence alignment of mutant MSV-VW genomes starting from the *Bam*HI restriction site of the movement protein. The file is in FASTA format and should be viewed using the program Mega5. Click here for file

Additional file 2 **Alignment of MSV-MatA-derived mutant genomes. **Full-genome sequence alignment of mutant MSV-MatA genomes starting from the *Bam*HI restriction site of the movement protein. The file is in FASTA format and should be viewed using the program Mega5. Click here for file

Additional file 3 **Alignment of mutated genomes obtained using the VWMPCPLIRMat chimaeric virus.** Full-genome sequence alignment of mutant VWMPCPLIRMat starting from the *Bam*HI restriction site of the movement protein. The file is in FASTA format and should be viewed using the program Mega5. Click here for file

Additional file 4 **Alignment of recombinant genomes obtained using the MatMPCPVW + VWMPCPMat parental pair. **Full-genome sequence alignment of recombinant MSV starting from the *Bam*HI restriction site of the movement protein. The file is in FASTA format and should be viewed using the program Mega5. Click here for file

Additional file 5 **Alignment of recombinant genomes obtained using the MatMPCPLIRVW + VWMPCPLIRMat parental pair. **Full-genome sequence alignment of recombinant MSV starting from the *Bam*HI restriction site of the movement protein. The file is in FASTA format and should be viewed using the program Mega5. Click here for file

Additional file 6 **Analysis of codons under selection in recombinant MSV, MSV-Mat and MSV-VW.** Description of genes and codons under positive or negative selection in the MVS-MatA, MSV-VW and VWMPCPLIRMat data sets. The file is in .xls format. Click here for file

Additional file 7 **Details of mutations in recombinant MSV and wild-type MSV. **Details on the viruses, the exact mutations observed and amino acid changes in those viruses obtained using the MatMPCPVW + VWMPCPMat and MatMPCPLIRVW + VWMPCPLIRMat parental pairs, VWMPCPLIRMat, MSV-MatA and MSV-VW. Note that additional detail relating to mutant viruses obtained in parallel and related evolutionary experiments (from the study of Monjane *et al*. [47]), yet not explained or discussed in this study, are included. The file is in .xls format. Click here for file

Additional file 8 **MSV-A genome annotated according to sites under negative and positive selection.** Distribution of regions under selection inferred using SLAC, REL and IFEL methods of selection analysis. Annotated also are the nucleotides that are mutated in the recombinant MSV data, as well as the MSV-MatA and MSV-VW data sets. Note that additional detail relating to mutant viruses obtained in parallel and related evolutionary experiments (from the study of Monjane *et al*. [47]), yet not explained or discussed in this study, are included. The file is in .doc format. Click here for file

Additional file 9 Mutation dynamics in regions of MSV-A genomes under specific types of selection. Click here for file

Additional file 10 **Variability of mutation dynamics in populations of recombinant and wild-type MSV genomes. **The file is in .xls format. Click here for file

Additional file 11 **Nucleotide mutation dynamics based on the observed number mutations occurring between pairs of nucleotides during short-term evolution studies using MSV and TYLCV.** The relative proportion of each mutation is expressed in each cell as the percentage of the total number of observed mutations under each experimental condition (the most commonly observed mutations are shown in bold). Cells in grey indicate instances in which no statistically significant (i.e. p < 0.05) difference was obtained between the observed and expected number of mutations; those in blue and red, however, indicate respectively instances where significantly less than expected, or significantly more than expected mutations were observed. The file is in .pdf format. Click here for file

## References

[B1] Shafer RW Schapiro JM HIV-1 drug resistance mutations: an updated framework for the second decade of HAART AIDS Rev 2008 10 2 67 84 18615118 PMC2547476

[B2] Chang MW Torbett BE Accessory mutations maintain stability in drug-resistant HIV-1 protease J Mol Biol 2011 410 756 760 10.1016/j.jmb.2011.03.038 21762813 PMC3139113

[B3] Leslie AJ Pfafferott KJ Chetty P Draenert R Addo MM Feeney M Tang Y Holmes EC Allen T Prado JG Altfeld M Brander C Dixon C Ramduth D Jeena P Thomas SA St John A Roach TA Kupfer B Luzzi G Edwards A Taylor G Lyall H Tudor-Williams G Novelli V Martinez-Picado J Kiepiela P Walker BD Goulder PJ HIV evolution: CTL escape mutation and reversion after transmission Nat Med 2004 10 3 282 289 10.1038/nm992 14770175

[B4] Cooreman MP Leroux-Roels G Paulij WP Vaccine- and hepatitis B immune globulin-induced escape mutations of hepatitis B virus surface antigen J Biomed Sci 2001 8 3 237 247 10.1007/BF02256597 11385295

[B5] Shackelton LA Parrish CR Truyen U Holmes EC High rate of viral evolution associated with the emergence of carnivore parvovirus PNAS 2005 102 2 379 384 10.1073/pnas.0406765102 15626758 PMC544290

[B6] Lalić J Cuevas JM Elena SF Effect of host species on the distribution of mutational fitness effects for an RNA virus PLoS Genet 2011 7 11 e1002378 10.1371/journal.pgen.1002378 22125497 PMC3219607

[B7] Jenner CE Tomimura K Ohshima K Hughes SL Walsh JA Mutations in Turnip mosaic virus P3 and cylindrical inclusion proteins are separately required to overcome two Brassica napus resistance genes Virology 2002 300 1 50 59 10.1006/viro.2002.1519 12202205

[B8] Montarry J Doumayrou J Simon V Moury B Genetic background matters: a plant-virus gene-for-gene interaction is strongly influenced by genetic contexts Mol Plant Pathol 2011 12 9 911 920 10.1111/j.1364-3703.2011.00724.x 21726391 PMC6640445

[B9] Lafforgue G Martínez F Sardanyés J de la Iglesia F Niu QW Lin SS Solé RV Chua NH Daròs JA Elena SF Tempo and mode of plant RNA virus escape from RNA interference-mediated resistance J Virol 2011 85 19 9686 9695 10.1128/JVI.05326-11 21775453 PMC3196453

[B10] Hébrard E Pinel-Galzi A Bersoult A Siré C Fargette D Emergence of a resistance-breaking isolate of rice yellow mottle virus during serial inoculations is due to a single substitution in the genome-linked viral protein VPg J Gen Virol 2006 87 5 1369 1373 10.1099/vir.0.81659-0 16603540

[B11] Flint SJ Enquist LW Racaniello VR Skalka AM Principles of Virology Molecular Biology, Pathogenesis and Control of Animal Viruses 2004 Washington: ASM

[B12] Pepin KM Domsic J McKenna R Genomic evolution in a virus under specific selection for host recognition Infect Genet Evol 2008 8 6 825 834 10.1016/j.meegid.2008.08.008 18804189

[B13] Rasmussen AL Racaniello VR Selection of rhinovirus 1A variants adapted for growth in mouse lung epithelial cells Virology 2011 420 2 82 88 10.1016/j.virol.2011.08.021 21943827 PMC3205939

[B14] Sanjuán R Mutational fitness effects in RNA and single-stranded DNA viruses: common patterns revealed by site-directed mutagenesis studies Phil Trans R Soc B 2010 365 1975 1982 10.1098/rstb.2010.0063 20478892 PMC2880115

[B15] Miralles R Gerrish PJ Moya A Elena SF Clonal interference and the evolution of RNA viruses Science 1999 285 1745 1747 10.1126/science.285.5434.1745 10481012

[B16] Escarmís C Perales C Domingo E Biological effect of Muller’s ratchet: distant capsid site can affect picornavirus protein processing J Virol 2009 83 13 6748 6756 10.1128/JVI.00538-09 19403672 PMC2698577

[B17] Duffy S Holmes EC Validation of high rates of nucleotide substitution in geminiviruses: phylogenetic evidence from East African cassava mosaic viruses J Gen Virol 2009 90 1539 1547 10.1099/vir.0.009266-0 19264617 PMC4091138

[B18] Harkins GW Delport W Duffy S Wood N Monjane AL Owor BE Donaldson L Saumtally S Triton G Briddon RW Shepherd DN Rybicki EP Martin DP Varsani A Experimental evidence indicating that mastreviruses probably did not co-diverge with their hosts Virol J 2009 6 104 10.1186/1743-422X-6-104 19607673 PMC2719613

[B19] Grigoras I Timchenko T Grande-Pérez A Katul L Vetten H-J Gronenborn B High variability and rapid evolution of a Nanovirus J Virol 2010 84 18 9105 9117 10.1128/JVI.00607-10 20592071 PMC2937624

[B20] Drake JW Rates of spontaneous mutation among RNA viruses Proc Natl Acad Sci USA 1993 90 4171 4175 10.1073/pnas.90.9.4171 8387212 PMC46468

[B21] Malpica JM Fraile A Moreno I Obies CI Drake JW García-Arenal F The rate and character of spontaneous mutation in an RNA virus Genetics 2002 162 1505 1511 12524327 10.1093/genetics/162.4.1505 PMC1462378

[B22] Garcia-Diaz M Bebenek K Multiple functions of DNA polymerases Crit Rev Plant Sci 2007 26 105 122 10.1080/07352680701252817 18496613 PMC2391090

[B23] Domingo E Holland JJ RNA virus mutations and fitness for survival Annu Rev Microbiol 1997 51 151 178 10.1146/annurev.micro.51.1.151 9343347

[B24] Walsh CP Xu GL Cytosine methylation and DNA repair Curr Topics Microbiol Immunol 2006 301 283 315 10.1007/3-540-31390-7_11 16570853

[B25] Duffy S Shackelton LA Holmes EC Rates of evolutionary change in viruses: patterns and determinants Nat Rev Gen 2008 9 267 276 10.1038/nrg2323 18319742

[B26] Chao L Rang CU Wong LE Distribution of spontaneous mutants and inferences about the replication mode of the RNA bacteriophage φ6 J Virol 2002 76 3276 3281 10.1128/JVI.76.7.3276-3281.2002 11884552 PMC136006

[B27] French R Stenger DC Evolution of wheat streak mosaic virus: dynamics of population growth within plants may explain limited variation Annu Rev Phytopathol 2003 41 199 214 10.1146/annurev.phyto.41.052002.095559 12730393

[B28] Duffy S Holmes EC Phylogenetic evidence for rapid rates of molecular evolution in the single-stranded DNA Begomovirus Tomato yellow leaf curl virus J Virol 2008 82 2 957 965 10.1128/JVI.01929-07 17977971 PMC2224568

[B29] van der Walt E Martin DP Varsani A Polston JE Rybicki EP Experimental observations of rapid Maize streak virus evolution reveal a strand-specific nucleotide substitution bias Virol J 2008 5 104 10.1186/1743-422X-5-104 18816368 PMC2572610

[B30] Rattray AJ Strathern JN Error-prone DNA polymerases: when making a mistake is the only way to get ahead Annu Rev Genet 2003 37 31 66 10.1146/annurev.genet.37.042203.132748 14616055

[B31] Chin MPS Lee S-K Chen J Nikolaitchik OA Powell DA Fivash MJ Jr Hu W-S Long-range recombination gradient between HIV-1 subtypes B and C variants caused by sequence differences in the dimerization initiation signal region J Mol Biol 2008 377 5 1324 1333 10.1016/j.jmb.2008.02.003 18314135 PMC2706499

[B32] Shcherbakov VP Plugina L Shcherbakova T Sizova S Kudryashova E On the mutagenicity of homologous recombination and double-strand break repair in bacteriophage DNA Repair 2011 10 16 23 10.1016/j.dnarep.2010.09.006 20951652

[B33] Martin DP Biagini P Lefeuvre P Golden M Roumagnac P Varsani A Recombination in eukaryotic single stranded DNA viruses Viruses 2011 3 9 1699 1738 21994803 10.3390/v3091699 PMC3187698

[B34] Patil BL Fauquet CM Cassava mosaic geminiviruses: actual knowledge and perspectives Mol Plant Pathol 2009 10 5 685 701 10.1111/j.1364-3703.2009.00559.x 19694957 PMC6640248

[B35] Navas-Castillo J Fiallo-Olivé E Sánchez-Campos S Emerging virus diseases transmitted by whiteflies Annu Rev Phytopathol 2011 49 219 248 10.1146/annurev-phyto-072910-095235 21568700

[B36] Martin DP Shepherd DN The epidemiology, economic impact and control of maize streak disease Food Security 2009 1 305 315 10.1007/s12571-009-0023-1

[B37] Lazarowitz SG Pinder AJ Molecular genetics of Maize streak virus UCLA Sym BI 1989 101 167 183

[B38] Hofer JM Dekker EL Reynolds HV Woolston CJ Cox BS Mullineaux PM Coordinate regulation of replication and virion sense gene expression in wheat dwarf virus Plant Cell 1992 4 213 223 1633494 10.1105/tpc.4.2.213 PMC160122

[B39] Collin S Fernández-Lobato M Gooding PS Mullineaux PM Fenoll C The two nonstructural proteins from wheat dwarf virus involved in viral gene expression and replication are retinoblastoma-binding proteins Virology 1996 219 324 329 10.1006/viro.1996.0256 8623550

[B40] Zhan X Richardson KA Haley A Morris BA The activity of the coat protein promoter of chloris striate mosaic virus is enhanced by its own and C1-C2 gene products Virology 1993 193 1 498 502 10.1006/viro.1993.1153 8438584

[B41] Hefferon KL Moon YS Fan Y Multi-tasking of nonstructural gene products is required for bean yellow dwarf geminivirus transcriptional regulation FEBS J 2006 273 19 4482 4494 10.1111/j.1742-4658.2006.05454.x 16972938

[B42] Boulton MI Buchholz WG Marks MS Markham PG Davies JW Specificity of Agrobacterium-mediated delivery of maize streak virus DNA to members of the Gramineae Plant Mol Biol 1989 12 31 40 10.1007/BF00017445 24272715

[B43] Lazarowitz SG Pinder AJ Damsteegt VD Rogers SG Maize streak virus genes essential for systemic spread and symptom development EMBO J 1989 8 1023 1032 16453874 10.1002/j.1460-2075.1989.tb03469.x PMC400910

[B44] Woolston CJ Reynolds HV Stacey NJ Mullineaux PM Replication of Wheat dwarf virus DNA in protoplasts and analysis of coat protein mutants in protoplasts and plants Nucleic Acids Res 1989 17 6029 6041 10.1093/nar/17.15.6029 2549505 PMC318258

[B45] Mullineaux PM Donson J Morris-Krsinich BAM Boulton MI Davies JW The nucleotide sequence of maize streak virus DNA EMBO J 1984 3 13 3063 3068 6526009 10.1002/j.1460-2075.1984.tb02258.x PMC557817

[B46] Willment JA Martin DP Palmer KE Schnippenkoetter WH Shepherd DN Rybicki EP Identification of long intergenic region sequences involved in maize streak virus replication J Gen Virol 2007 88 1831 1841 10.1099/vir.0.82513-0 17485545

[B47] Monjane AL van der Walt E Varsani A Rybicki EP Martin DP Recombination hotspots and host susceptibility modulate the adaptive value of recombination during maize streak virus evolution BMC Evol Biol 2011 11 350 10.1186/1471-2148-11-350 22136133 PMC3280948

[B48] Martin DP Lefeuvre P Varsani A Hoareau M Semegni J-Y Dijoux B Vincent C Reynaud B Lett J-M Complex recombination patterns arising during Geminivirus coinfections preserve and demarcate biologically important intra-genome interaction networks PLoS Pathog 2011 7 9 e1002203 10.1371/journal.ppat.1002203 21949649 PMC3174254

[B49] Willment JA Martin DP van der Walt E Rybicki EP Biological and genomic sequence characterization of Maize streak virus isolates from wheat Phytopathology 2002 92 81 86 10.1094/PHYTO.2002.92.1.81 18944143

[B50] Martin DP Willment JA Billharz R Velders R Odhiambo B Njuguna J James D Rybicki EP Sequence diversity and virulence in Zea mays of Maize streak virus isolates Virology 2001 288 247 255 10.1006/viro.2001.1075 11601896

[B51] Martin DP Rybicki EP Investigation of maize streak virus pathogenicity determinants using chimaeric genomes Virology 2002 300 180 188 10.1006/viro.2002.1458 12350349

[B52] Martin DP Willment JA Rybicki EP Evaluation of maize streak virus pathogenicity in differentially resistant Zea mays genotypes Phytopathology 1999 89 8 695 700 10.1094/PHYTO.1999.89.8.695 18944683

[B53] Inoue-Nagata AK Albuquerque LC Rocha WB Nagata T A simple method for cloning the complete begomovirus genome using the bacteriophage Q29 DNA polymerase J Virol Methods 2004 116 209 211 10.1016/j.jviromet.2003.11.015 14738990

[B54] Shepherd DN Martin DP Lefeuvre P Monjane AL Owor BE Rybicki EP Varsani A A protocol for the rapid isolation of full geminivirus genomes from dried plant tissue J Virol Methods 2008 149 97 102 10.1016/j.jviromet.2007.12.014 18280590

[B55] Sambrook J Fritsch EF Maniatis T Molecular cloning: A laboratory manual 1989 2 Cold Spring Harbor, New York: Cold Spring Harbor Laboratory

[B56] Owor BE Shepherd DN Taylor NJ Edema R Monjane AL Thomson JA Martin DP Varsani A Successful application of FTA Classic Card technology and use of bacteriophage Q29 DNA polymerase for large-scale field sampling and cloning of complete maize streak virus genomes J Virol Methods 2007 140 100 105 10.1016/j.jviromet.2006.11.004 17174409

[B57] Kosakovsky Pond SL Frost SDW Not so different after all: A comparison of methods for detecting amino acid sites under selection Mol Biol Evol 2005 22 5 1208 1222 10.1093/molbev/msi105 15703242

[B58] Kosakovsky Pond SL Frost SDW Datamonkey: rapid detection of selective pressure on individual sites of codon alignments Bioinformatics 2005 21 2531 2533 10.1093/bioinformatics/bti320 15713735

[B59] Felsenstein J Evolutionary trees from DNA sequences: a maximum likelihood approach J Mol Evol 1981 17 368 376 10.1007/BF01734359 7288891

[B60] Martin DP Lemey P Lott M Moulton V Posada D Lefeuvre P RDP3: a flexible and fast computer program for analyzing recombination Bioinformatics 2010 26 2462 2463 10.1093/bioinformatics/btq467 20798170 PMC2944210

[B61] García-Andrés S Accotto GP Navas-Castillo J Moriones E Founder effect, plant host, and recombination shape the emergent population of begomoviruses that cause the tomato yellow leaf curl disease in the Mediterranean basin Virology 2007 359 2 302 312 10.1016/j.virol.2006.09.030 17070885

[B62] Fondong V Chen K Genetic variability of East African cassava mosaic Cameroon virus under field and controlled environment conditions Virol 2011 413 275 282 10.1016/j.virol.2011.02.024 21429548

[B63] Pita JS De Miranda JR Schneider WL Roossinck MJ Environment determines fidelity for an RNA virus replicase J Virol 2007 81 9072 9077 10.1128/JVI.00587-07 17553888 PMC1951419

[B64] Shepherd DN Martin DP McGivern DR Boulton MI Thomson JA Rybicki EP A three-nucleotide mutation altering the Maize streak virus Rep pRBR-interaction motif reduces symptom severity in maize and partially reverts at high frequency without restoring pRBR–Rep binding J Gen Virol 2005 86 803 813 10.1099/vir.0.80694-0 15722543

[B65] Shepherd DN Martin DP Varsani A Thomson JA Rybicki EP Klump HH Restoration of native folding of single-stranded DNA sequences through reverse mutations: An indication of a new epigenetic mechanism Arch Biochem Biophys 2006 453 108 122 10.1016/j.abb.2005.12.009 16427599

[B66] Ge L Zhang J Zhou X Li H Genetic structure and population variability of Tomato yellow leaf curl China virus J Virol 2007 81 11 5902 5907 10.1128/JVI.02431-06 17376922 PMC1900275

[B67] Arguello-Astorga G Ascencio-Ibáñez JT Dallas MB Orozco BM Hanley-Bowdoin L High-frequency reversion of geminivirus replication protein mutants during infection J Virol 2007 81 20 11005 11015 10.1128/JVI.00925-07 17670823 PMC2045516

[B68] Domingo-Calap P Cuevas JM Sanjuán R The fitness effects of random mutations in single-stranded DNA and RNA bacteriophages PLoS Genet 2009 11 e1000742 19956760 10.1371/journal.pgen.1000742 PMC2776273

[B69] Liu H Boulton MI Thomas CL Prior DA Oparka KJ Davies JW Maize streak virus coat protein is karyophyllic and facilitates nuclear transport of viral DNA Molec Plant Microbe In 1999 12 894 900 10.1094/MPMI.1999.12.10.894 10517029

[B70] Wright EA Heckel T Groenendijk J Davies JW Boulton MI Splicing features in maize streak virus virion- and complementary-sense gene expression Plant J 1997 12 1285 1297 10.1046/j.1365-313x.1997.12061285.x 9450342

[B71] Boulton MI King DI Donson J Davies JW Point substitution in a promoter-like region and the V1 gene affect the host range and symptoms of maize streak virus Virology 1991 183 1 114 121 10.1016/0042-6822(91)90124-T 2053276

[B72] Eagle PA Hanley-Bowdoin L cis elements that contribute to Geminivirus transcriptional regulation and the efficiency of DNA replication J Virol 1997 71 9 6947 6955 9261423 10.1128/jvi.71.9.6947-6955.1997 PMC191979

[B73] Boulton MI King DI Markham PG Pinner MS Davies JW Host range and symptoms are determined by specific domains of the maize streak virus genome Virology 1991 181 1 312 318 10.1016/0042-6822(91)90497-Y 1994579

[B74] Lefeuvre P Lett JM Reynaud B Martin DP Avoidance of protein fold disruption in natural virus recombinants PLoS Pathog 2007 3 11 e181 10.1371/journal.ppat.0030181 18052529 PMC2092379

[B75] Varsani A Shepherd DN Monjane AL Owor BE Erdmann JB Rybicki EP Peterschmitt M Briddon RW Markham PG Oluwafemi S Windram OP Lefeuvre P Lett J-M Martin DP Recombination, decreased host specificity and increased mobility may have driven the emergence of maize streak virus as an agricultural pathogen J Gen Virol 2008 89 2063 2074 10.1099/vir.0.2008/003590-0 18753214 PMC2886952

[B76] Padidam M Beachy RN Fauquet CM Classification and identification of geminiviruses using sequence comparisons J Gen Virol 1995 76 249 263 10.1099/0022-1317-76-2-249 7844548

[B77] Delatte H Holota H Moury B Reynaud B Lett J-M Peterschmitt M Evidence for a founder effect after introduction of Tomato Yellow Leaf Curl Virus–Mild in an insular environment J Mol Evol 2007 65 112 118 10.1007/s00239-007-0005-x 17609843

[B78] Isnard M Granier M Frutos R Reynaud B Peterschmitt M Quasispecies nature of three maize streak virus isolates obtained through different modes of selection from a population used to assess response to infection of maize cultivars J Gen Virol 1998 79 3091 3099 9880027 10.1099/0022-1317-79-12-3091

[B79] Urbino C Thébaud G Granier M Blanc S Peterschmitt M A novel cloning strategy for isolating, genotyping and phenotyping genetic variants of geminiviruses Virol J 2008 5 135 10.1186/1743-422X-5-135 18976479 PMC2585570

[B80] Elena SF Miralles R Cuevas JM Turner PE Moya A The two faces of mutation: extinction and adaptation in RNA viruses IUBMB Life 2000 49 5 9 10772334 10.1080/713803585

[B81] Umemura T Tanaka Y Kiyosawa K Alter HJ Wai-Kuo Shih J Observation of positive selection within hypervariable regions of a newly identified DNA virus (SEN virus) FEBS Lett 2002 510 171 174 10.1016/S0014-5793(01)03258-6 11801248

[B82] Pu Y Kikuchi A Moriyasu Y Tomaru M Jin Y Suga H Hagiwara K Akita F Shimizu T Netsu O Suzuki N Uehara-Ichiki T Sasaya T Wei T Li Y Omura T Rice dwarf viruses with dysfunctional genomes generated in plants are filtered out in vector insects: Implications for the origin of the virus J Virol 2011 85 6 2975 2979 10.1128/JVI.02147-10 21191024 PMC3067918

[B83] Brough CL Gardiner WE Inamdar NM Zhang XY Ehrlich M Bisaro DM DNA methylation inhibits propagation of tomato golden mosaic virus DNA in transfected protoplasts Plant Mol Biol 1992 18 4 703 712 10.1007/BF00020012 1558945

[B84] Roossinck MJ Mechanisms of plant virus evolution Annu Rev Phytopathol 1997 35 191 209 10.1146/annurev.phyto.35.1.191 15012521

[B85] Frederico LA Kunkel TA Shaw BR A sensitive genetic assay for the detection of cytosine deamination: determination of rate constants and the activation energy Biochemistry 1990 29 10 2532 2537 10.1021/bi00462a015 2185829

[B86] Caulfield JL Wishnok JS Tannenbaum SR Nitric oxide induced deamination of cytosine and guanine in deoxynucleosides and oligonucleotides J Biol Chem 1998 273 12689 12695 10.1074/jbc.273.21.12689 9582291

[B87] Stasolla C Katahira R Thorpe TA Ashihara H Purine and pyrimidine nucleotide metabolism in higher plants J Plant Physiol 2003 160 11 1271 1295 10.1078/0176-1617-01169 14658380

[B88] Xia X Yuen KY Differential selection and mutation between dsDNA and ssDNA phages shape the evolution of their genomic AT percentage BMC Genet 2005 11 6 20 15823202 10.1186/1471-2156-6-20 PMC1087484

[B89] Zrenner R Stitt M Sonnewald U Boldt R Pyrimidine and purine biosynthesis and degradation in plants Annu Rev Plant Biol 2006 57 805 836 10.1146/annurev.arplant.57.032905.105421 16669783

