# Supplementary material for: Adaptive evolution by recombination is not associated with increased mutation rates in Maize streak virus
Source: BMC Evol Biol. 2012 Dec 27;12:252. doi: 10.1186/1471-2148-12-252 (PMC3556111; doi:10.1186/1471-2148-12-252)
Supplement: Additional file 8 — MSV-A genome annotated according to sites under negative and positive selection. Distribution of regions under selection inferred using SLAC, REL and IFEL methods of selection analysis. Annotated also are the nucleotides that are mutated in the recombinant MSV data, as well as the MSV-MatA and MSV-VW data sets. Note that additional detail relating to mutant viruses obtained in parallel and related evolutionary experiments (from the study of Monjane et al. [47]), yet not explained or discussed in this study, are included. The file is in .doc format. [file 1471-2148-12-252-S8.doc]

Origin of (+) strand replication

AT -tracts

MSV-A1 TAATATTACCGCGCCTTCTTTT.CCTGCGAGGGCCCGGTAGGGCCCGAGCGATTTGATGTAAAGCTTGGTCCTGCTTTGTATGATTTATCTAAAGCAGCC 99

MSV-A2 TAATATTACCGCGCCTTCTTTT.CCTGCGAGGGCCCGGTAGGGCCCGAGCGATTTGATGTAAAGtTTGGTCCTGCTTTGTATGATTTATCTAAAGCAGCC 99

MSV-A3 TAATATTACCGCGCCTTCTTTT.CCTGCGAGGGCCCGGTAGGGCCCGAGCGATTTGATGTAAAGCTTGGTCCTGCTTTGTATGATTTATCTAAAGCAGCC 99

MSV-A4 TAATATTACCGCGCCTTCTTTT.CCTGCGAGGGCCCGGTAGGGaCCGAGCGcTTTGATtTAAAGCcTGGTtCTGCTTTGTATGATTTATCTAAAGCAGCC 99

MSV-A6 TAATATTACCGCGCCTTCTTTT.CCTGCGAGGtCCCGGTAGGGaCCGAGCGATTTGATtTAAAGCTTGGTCCTGCTTTGTATGATTTATCTAAAGCAGCC 99

MSV-B TAATATTACCGCGCCTTCTTTT.tCTGCGAGGGCCCGa.AGGGaCCGAGCGATTTGATtTAAAGtTcGGTtCTGCTTTGTcTGATTTATCTAAAGCAGCC 98

Consensus taatattaccgcgccttctttt ctgcgagg cccg aggg ccgagcg tttgat taaag ggt ctgctttgt tgatttatctaaagcagcc

SLAC

REL

IFEL

MP Initiation codon

TATA box

*MP* gene

AT -tracts

MSV-A1 CATTCTAAAGAAACCGGTCCCGGGC.ACTATAAATTGCCTAACAAGTGCGATTCATTCATGGATCCACAGAACGCCCTGTATTATCAGCCGCGGGTACCC 198

MSV-A2 CATTCTAAAGAAtCCGGTCCCGGtC.ACTATAAATTGCCTAACAAGTGCGATTCATTCATGGATCCACAGAACGCCCTGTATTATCAGCCGCGGGTACCC 198

MSV-A3 CAaTCTAAAGAAACCGGTCCCGGGC.ACTATAAATTGCCTAACAAGTGCGATTCATTCATGGATCCACAGAACGCCCTGTATTATCAGCCGCGGGTACCC 198

MSV-A4 CAaTCTAAAGAAACCGGTCCCGGGC.ACTATAAATTGCCTAACAAGTGCGATTCATTCATGGATCCACAGAACGCCCTGTATTATCAGCCGCGGGTACCC 198

MSV-A6 tAaTCTAAAGAAtCCGGTCCCGGGC.ACTATAAATTGtCTAACAAGTGCGATTCAcTCATGGATCCACAGAACtCCtTGTATgtaCAGCCGCGGGTACCC 198

MSV-B CAaTCTAAAGAAACCGGTCCCctGCgACTATAAATTGtCTcACAAGTGCGATTCATTCATGGATCCACAGAACtCCtTcTtTgtaCAGCCGCGGGTACCC 198

Consensus a tctaaagaa ccggtccc c actataaattg ct acaagtgcgattca tcatggatccacagaac cc t t t cagccgcgggtaccc

SLAC

REL

IFEL

Donor site

T-tracts

Intron

Cryptic donor site

MSV-A1 ACAGCAGCTCCGGCATCCGCAGGAGTGCCGTGGAGTCGCGTAGGCGAGGTAGCTATTTTGAGCTTTGTTGCACTGATTTGCTTTTACCTGCTTTACCTTT 298

MSV-A2 ACAGCAGCTCCGaCATCCGgAGGAGTGCCGTGGAGTCGCGTAGGCGAGGTAGCTATTTTGAGCTTTGTTGCAtTGATTTGCTTTTACCTGCTTTACCTTT 298

MSV-A3 ACAGCAGCTCCGaCATCCGgAGGAGTGCCGTGGAGTCGCGTAGGCGAGGTAGCTATTTTGAGCTTTGTTGCAtTGATTTGCTTTTACCTGCTTTACCTTT 298

MSV-A4 ACAGCAGCTCCGaCATCCGgAGGAGTGCCGTGGAGTCGCGTAGGCGAGGTAGCTATTTTGAGCTTTGTTGCAtTGATTTGCTTTTACCTGCTTTACCTTT 298

MSV-A6 ACAGCAGCTCCGaCcTCCGCAGGAGTGCCGTGGAGTCGCGTAGGCGAGGTAGCTgTTTTGAGCTTTGTTGCAtTGATTTGCTTTTACCTGCTTTACCTTT 298

MSV-B ACAGCAGCTCCGaCATCCGgAGGAGTGtCGTGGAGTCGCGTAGGCGAGGTAGCTATTTTGAGCTTTGTTGCttTGATTatCTTTTACCTGCTTTACCTTT 298

Consensus acagcagctccg c tccg aggagtg cgtggagtcgcgtaggcgaggtagct ttttgagctttgttgc tgatt cttttacctgctttaccttt

SLAC

REL

IFEL

Branch point

Acceptor site

Cryptic acceptor site

MSV-A1 GGGTGCTGAGAGACCTTATCTTAGTTCTGAAGGCTCGACAAGGCAGATCCACGGAGGAGCTGATATTTGGAGGACAAGCTGTGGATAGGAGCAACCCTAT 398

MSV-A2 GGGTGCTGAGAGACCTTATCTTAGTTCTGAAGGCTCGACAAGGCAGATCCACGGAGGAGCTGATATTTGGtGGACAAGCTGTGGATAGGAGCAACCCTAT 398

MSV-A3 GGGTGCTGAGAGACCTTATCTTAGTTCTGAAGGCTCGACAAGGCAGATCCACGGAGGAGCTGATATTTGGAGGACAAGCTGTGGATAGGAGCAACCCTAT 398

MSV-A4 GGGTGCTGAGAGACCTTATCTTAGTTCTGAAGGCTCGACAAGGCAGATCCACGGAGGAGCTGATATTTGGtGGACAAGCTGTGGATAGGAGCAACCCTAT 398

MSV-A6 GGGTGCTGAGAGACCTTATCTTAGTTCTGAAGGCTCGcCAAGGCAGATCCACGGAGGAGCTGATATTTGGtGGACAAGCTGTGGATAGGAGCAACCCTAT 398

MSV-B GGGTGCTGAGAGAtCTTATCTTAGTTCTGAAGGCTCGACAAGGCAGATCCACGGAGGAGCTGATATTTGGAatACAAGCTGTGGATAGGAGCAACCCTAT 398

Consensus gggtgctgagaga cttatcttagttctgaaggctcg caaggcagatccacggaggagctgatatttgg acaagctgtggataggagcaaccctat

SLAC

REL

IFEL

MP stop codon

CP initiation codon

CP gene

MSV-A1 CCCTAATCTACCAGCACCACCAAGTCAGGGCAATCCCGGGCCATTTGTTCCAGGCACGGGATAAGCATTCAGCCATGTCCACGTCCAAGAGGAAGCGGGG 498

MSV-A2 CCCTAATaTACCAGCACCACCAAGTCAGGGCAATCCCGGGCCATTTGTTCCAGGCACGGGATAAGCATTCAGCCATGTCCACGTCCAAGAGGAAGCGGGG 498

MSV-A3 CCCTAATaTACCAGCACCACCAAGTCAGGGCAATCCCGGGCCATTTGTTCCAGGCACGGGATAAGCAaTCAGCCATGTCCACGTCCAAGAGGAAGCGGGG 498

MSV-A4 CCCTAATCTACCttCACCACCAAGTCAGGGCAATCCCGGGCCATTTGTTCCAGGCACGGGATAAGCAaTCAGCCATGTCCACGTCCAAGAGGAAGCGGGG 498

MSV-A6 CCCTAATaTACCAGCACCACCAAGTCAGGGtAATCCCGGGCCATTTGTTCCAGGCACGGGATAAGCAaTCAGCCATGTCCACGTCCAAGAGGAAGCGGGG 498

MSV-B CCCTAATacACagGCACCACCAAtTCAGGGgAATCCCGGGCCgTTcGTTCCAGGCACGGGATAAGCAaTCAGCCATGTCtACtTCCAAGAGGAAaCGGGG 498

Consensus ccctaat ac caccaccaa tcaggg aatcccgggcc tt gttccaggcacgggataagca tcagccatgtc ac tccaagaggaa cgggg

SLAC

REL

IFEL

MSV-A1 AGATGATTCGAATTGGAGTAAGCGGGTGCCTAAGAAGAAGCCCTCTTCAGCTGGGCTGAAGAGGGCTGGAAGCAAGGCCGATAGGCCATCCCTCCAAATC 598

MSV-A2 AGATGATTCGAATTGGAGTAAGCGGGTGaCTAAGAAGAAGCCtTCTTCAGCTGGGCTGAAGAGGGCTGGcAGCAAGGCCGATAGGCCATCCCTgCAAATC 598

MSV-A3 AGATGATgCGAATTGGAGTAAGCGGGTGtCTAAGAAGAAGCCtTCTTCAGCTGGGCTGAAGAGGGCTGGAAGCAAGGCCGATAGGCCATCCCTCCAAATC 598

MSV-A4 AGATGATTCGAATTGGAaTAAGCGGGTGCCTAAGAAGAAGCCaTCTTCAGCTGGGCTGAAGAGGGCTGGAAGCAAGGCCGATAGGCCATCCCTCCAAATC 598

MSV-A6 AGATGATTCGAATTGGAaTAAGCGGGTttCTAAGAAGAAGCCtTCTTCAGCTGGGCTGAAGAGGGCTGGcAGCAAGGCCGATAGGCCATCCCTCCAAATC 598

MSV-B AGATGATTCGAAcTGGAaTAAGCGGacGaCTAAGAAGAAGCCtTCTTCAGCTGGtCTGAAGAaGGCTGGAAGCAAGGCCGAaAGGCCATCCCTtCAgATC 598

Consensus agatgat cgaa tgga taagcgg ctaagaagaagcc tcttcagctgg ctgaaga ggctgg agcaaggccga aggccatccct ca atc

SLAC

REL

IFEL

MSV-A1 CAGACACTCCAGCATGCTGGGACCACCATGATAACTGTCCCATCCGGAGGAGTATGTGACCTCATCAACACCTATGCCCGAGGATCTGACGAGGGCAACC 698

MSV-A2 CAGACACTCCAGCAcGCTGGGACCACCATGATAACgGTCCCcTCCGGAGGAGTATGTGACCTCATCAACACCTATGCCCGAGGATCTGACGAGGGCAACC 698

MSV-A3 CAGACACTCCAGCATGCTGGGACCACCATGATAACgGTCCCcTCCGGAGGAGTATGTGACCTCATCAACACCTATGCCCGAGGATCTGACGAGGGCAACC 698

MSV-A4 CAGACACTCCAGCATGCTGGGACCACCATGATAACTGTCCCATCCGGAGGAGTATGTGACCTCATCAACACCTATGCCCGAGGATCTGACGAGGGCAACC 698

MSV-A6 CAGACACTgCAGCATGCcGGGACCACCATGATAACgGTCCCcTCCGGAGGcGTATGTGACCTCATCAACACCTATGCCCGAGGATCTGACGAGGGCAACC 698

MSV-B CAGACACTCCAGCATGCTGGGtCCACCATGATAACaGTCCCcTCCGGAGGAGTATGTGACCTCATCAACACCTATGCCCGAGGATCaGACGAGGGCAACC 698

Consensus cagacact cagca gc ggg ccaccatgataac gtccc tccggagg gtatgtgacctcatcaacacctatgcccgaggatc gacgagggcaacc

MSV-A1 GCCACACCAGCGAGACTCTCACGTACAAGATCGCCATCGACTACCACTTCGTTGCCGACGCGGCAGCCTGCCGCTACTCCAACACCGGAACCGGTGTAAT 798

MSV-A2 GCCACACCAGCGAGACTCTgACGTACAAGATCGCCATCGACTACCACTTCGTTGCCGACGCGGCAGCCTGCCGCTACTCCAACACCGGtACCGGTGTAAT 798

MSV-A3 GCCACACCAGCGAGACTCTgACGTACAAGATtGCCgTCGACTACCACTTCGTTGCCGACGCGGCAGCCTGCCGCTACTCCAACACCGGAACCGGTGTAAT 798

MSV-A4 GCCACACCAGCGAGACTCTgACGTACAAGATCGCCgTCGACTACCACTTCGTTGCCGACGCGGCtGCCTGCCGCTACTCCAACACCGGAACCGGTGTAAT 798

MSV-A6 GCCACACCAGCGAGACTCTtACGTACAAGATCGCCATCGACTACCACTTCGTTGCtGACGCGGCAGCCTGCCGgTACTCCAACACCGGAACCGGTGTcAT 798

MSV-B GCCACACCAGCGAGACTCTgACGTACAAGATtGCCgTCGACTACCACTTCGTTGCaGACtCGcaAGCCTGCaagTAtTCtAACACCGGgACCGGTGTgAT 798

Consensus gccacaccagcgagactct acgtacaagat gcc tcgactaccacttcgttgc gac cg gcctgc ta tc aacaccgg accggtgt at

MSV-A1 GTGGCTGGTGTATGACACCACTCCCGGCGGACAAGCTCCGACCCCGCAAACTATATTTGCCTACCCTGACACGCTAAAAGCGTGGCCGGCCACATGGAAG 898

MSV-A2 GTGGCTGGTGTATGACACCACTCCCGGCGGACAAGCTCCGACCCCGCAAACTATATTTGCCTACCCTGACACGCTgAAAGCGTGGCCGGCCACATGGAAa 898

MSV-A3 GTGGCTGGTGTATGACACCACTCCCGGCGGACAAGCTCCGACCCCGCAAACTATATTTGCCTACCCTGACACGCTAAAAGCGTGGCCGGCCACATGGAAa 898

MSV-A4 GTGGCTGGTGTATGACACCACTCCCGGCGGACAAGCTCCGACCCCGCAAACTATATTTGCCTACCCTGACACGCTAAAAGCGTGGCCGGCCACATGGAAa 898

MSV-A6 GTGGCTGGTGTATGACACCACTCCCGGCGGACAAGCTCCGACCCCcCAgACaATATTTGCCTACCCgGACACcCTcAAAGCcTGGCCGGCgACATGGAAG 898

MSV-B GTGGCTGGTGTAcGACACCACTCCCGGCGGACAAGCgCCGACCCCaCAAACcATATTTGCaTAtCCgGACACGCTgAAAGCtTGGCCGGCaACATGGAAa 898

Consensus gtggctggtgta gacaccactcccggcggacaagc ccgacccc ca ac atatttgc ta cc gacac ct aaagc tggccggc acatggaa

MSV-A1 GTGAGCCGGGAGCTGTGTCATCGCTTCGTGGTGAAACGGCGATGGTTGTTCAACATGGAGACCGACGGGCGGATTGGTTCGGACATTCCTCCCTCGAATG 998

MSV-A2 GTGAGCCGGGAGCTGTGTCATCGCTTCGTGGTGAAACGGCGATGGTTGTTCAACATGGAGACCGACGGGCGcATTGGTTCGGAtATTCCTCCCTCGAATG 998

MSV-A3 GTGAGCCGGGAGCTGTGTCATCGCTTCGTGGTGAAACGGCGATGGTTGTTCAACATGGAGACCGACGGtCGGATTGGTTCGGAtATTCCTCCaTCGAATG 998

MSV-A4 GTGAGCCGGGAGCTGTGTCATCGCTTCGTGGTGAAACGGCGATGGTTGTTCAACATGGAGACCGACGGtCGGATTGGTTCGGAtATcCCTCCCTCGAATa 998

MSV-A6 GTGAGCCGGGAGCTGTGTCATCGCTTCGTGGTGAAACGGCGtTGGTTGTTCAACATGGAGACCGACGGGCGGATTGGTTCGGACATTCCTCCaTCGAATG 998

MSV-B GTGAGCCGGGAGCTGTGTCATCGCTTCGTGGTGAAACGGCGATGGTTGTTCAACATGGAGACCGACGGtCGaATTGGTTCGGACATcCCTCCtTCGAATG 998

Consensus gtgagccgggagctgtgtcatcgcttcgtggtgaaacggcg tggttgttcaacatggagaccgacgg cg attggttcgga at cctcc tcgaat

MSV-A1 CAAGTTGGAAGCCTTGCAAGCGCAACATCTACTTCCACAAGTTCACGAGCGGGTTGGGAGTGAGAACGCAGTGGAAGAATGTAACGGACGGAGGAGTTGG 1098

MSV-A2 CAAGTTGGAAGCCTTGCAAGCGCAACATCTACTTCCACAAGTTCACGAGtGGGTTGGGAGTGAGAACGCAGTGGAAGAATGTAACGGACGGAGGAGTTGG 1098

MSV-A3 CAAGTTGGAAGCCTTGCAAGCGCAACATCTACTTCCACAAGTTCACGAGCGGGTTGGGAGTGAGAACGCAGTGGAAGAATGTAACGGACGGAGGAGTTGG 1098

MSV-A4 CAAGTTGGAAGCCTTGCAAGCGCAACATCTACTTCCACAAGTTCACGAGtGGGTTGGGAGTGAGAACGCAGTGGAAGAATGTAACGGACGGAGGAGTTGG 1098

MSV-A6 CAAGTTGGAAGCCTTGCAAGCGCAACATCTACTTCCACAAGTTCACGAGtGGGTTGGGAGTGAGAACGCAGTGGAAGAATGTAACGGACGGAGGAGTTGG 1098

MSV-B CAAGTTGGAAGCCgTGCAAGCGCAACATCTACTTCCACAAGTTCACGAGtGGGTTGGGAGTGAGAACGCAGTGGAAGAATGTAACGGACGGAGGAGTTGG 1098

Consensus caagttggaagcc tgcaagcgcaacatctacttccacaagttcacgag gggttgggagtgagaacgcagtggaagaatgtaacggacggaggagttgg

MSV-A1 TGCCATCCAGAGAGGAGCGTTGTACATGGTCATTGCCCCCGGCAATGGTCTTACATTTACTGCCCATGGGCAGACCCGTCTGTACTTTAAGAGTGTTGGC 1198

MSV-A2 TGCCATCCAGAGAGGAGCGcTGTACATGGTCATTGCCCCCGGCAATGGcCTTACtTTTACTGCCCATGGGCAGACCCGTCTGTACTTTAAGAGTGTTGGC 1198

MSV-A3 TGCCATCCAGAGAGGAGCccTGTACATGGTCATTGCCCCCGGCAATGGTCTTACtTTTACTGCCCATGGGCAGACCCGTCTGTACTTTAAGAGTGTTGGC 1198

MSV-A4 TGCCATCCAGAGAGGAGCtcTGTACATGGTCATTGCCCCaGGCAATGGcCTTACtTTTACTGCCCATGGGCAGACCCGTCTGTACTTTAAGAGTGTTGGC 1198

MSV-A6 TGCCATCCAGAGAGGAGCtTTGTACATGGTCATTGCCCCCGGCAATGGcCTTACATTTACTGCCCATGGGCAGACCCGTCTGTACTTTAAGAGTGTTGGg 1198

MSV-B TGCgATaCAGAGAGGAGCtcTGTACATGGTCATTGCCCCtGGCAATGGcCTTACATTTACTGCCCATGGGCAGACCCGTCTGTACTTTAAGAGTGTcGGC 1198

Consensus tgc at cagagaggagc tgtacatggtcattgcccc ggcaatgg cttac tttactgcccatgggcagacccgtct tactttaagagtgt gg

V- sense transcript polyadenylation signal

Primer binding site

CP termination codon

C- sense transcript polyadenylation signal

MSV-A1 AACCAGTAATGAATAAAAACTCCCGTTTTATTATATCTGATGAATGCTGAAAGCTTACATTAATATGTCGTGCGATGGCACGAAAAAACACACGCAATCA 1298

MSV-A2 AACCAGTAATGAAT.AAAACgCCgtTTTTATTATATCTGATGAATGCTGAAAGCTTACATTAATATGTCGTGCGATGGCACG.AAAAACACACaCAATCA 1296

MSV-A3 AACCAGTAATGAAT.AAAACgCCgtTTTTATTATAgCTGATGAATGCTGAAAGCTTACATTAATATGTCGTGCGATGGCACG.AAAAACACACGCAATCA 1296

MSV-A4 AACCAGTAATGAATAAAAACTCCCGTTTTATTATATtTGATGAATGCTGAAAGCTTACATTAATATGTCGTGCGATGGCACGAAAAAACACACGCAAaCA 1298

MSV-A6 AACCAGTAATGAAT.AAAACTCCgtTTTTATTATATCTcATGAATGCTGAAAGCTTACATTAATATGTCGTGCGATGGCACG.AAAAACACACaCAATCA 1296

MSV-B AACCAGTAATGAATAAAAACTCCCGTTTTATTATATCTGATGAATGCTcAAAGCTTACATTAATATGTCGTGCGATGGCACG.AAAAACACACtggAaCg 1297

Consensus aaccagtaatgaat aaaac cc ttttattata t atgaatgct aaagcttacattaatatgtcgtgcgatggcacg aaaaacacac a c

Sequence conserved in MSV subgenomic DNAs

Primer binding site

C2 termination codon

MSV-A1 ATACAGGGGGGTAGTAGGCGGGCGGCTAAGGGTGGTGCTCGGCGG.GCAGAACATCGAAAAATCAAGATCTATCTGAA.TGTACTGCCTCCGTAGGAGGC 1396

MSV-A2 ATACAGGGGGGTAGTcGGCGGGCGGCTAAGGGTGGTGCTCGGCGG.GCAGAACATCGAAAAATCAAGATCTATCTGAA.TGTACTGCCTCCGTAGGAGGC 1394

MSV-A3 ATACAGGGGGGTAGTcGGCGGGCGGCTAAGGGTGGTGCTCGGCGG.GCAaAACATCGAAAAATCAAGATCTATCTGAA.TGTACTGCCTCCGTAGGAGGC 1394

MSV-A4 ATACAGGGGGGTAGTcGGCGGGCGGCTAAGGGTGGTGCTCGGCGG.GCAGAACATCGAAAAATCAAGATCTATaTGAAtTacACTtCCTCCGTAGGAGGa 1397

MSV-A6 ATACAGGGGGGTAGTcGGCGGGCGGCTAAGGGTGGTGCTCGGCGG.GCAaAACATCGAAAAATCAAGATgTATCTGAA.TGTACTGCCTCCGTAGGAGGC 1394

MSV-B cagCcccGGGcTAaagacCGGGtctCaAgaG.....aCcCtGCGataCAaAACATCGAAAAAaCAAGATCTATaTGAAtTacACTtCCTCCGTAGGAGGa 1392

Consensus c ggg ta cggg c a g c c gcg ca aacatcgaaaaa caagat tat tgaa t act cctccgtaggagg

Stem sequence

Loop sequence

MSV-A1 AGCTCAGGGGGAGAATACCACTTCTCCCCCGGCGACATAATGTAAATGACGCAGTTTGCCTCGAAATACTCCAGCTGCCCTGGAGTCATTTCCTTCATCC 1496

MSV-A2 AGCTCAGGGGGAGAATACCAtTTCTCCCCCGGCGACATAATGTAAATGAtGCAGTTTGCCTCGAAATACTCCAGCTGCCCTGGAGTCATTTCCTTCATCC 1494

MSV-A3 AGCTCAGGGGGAGAATACCACTTCTCCCCCGGCGACATAATGTAAATGAtGCAGTTTGCCTCGAAATAaTCCAGCTGCCCTGGAtTCATTTCCTTCATCC 1494

MSV-A4 AGCaCAGGGGGAGAATACCACTTCTCCCCCGGCGACATAATGTAAATGACGCAGTTTGCCTCGAAATACTCCAGCTGCCCTGGAGTCATTTCCTTCATCC 1497

MSV-A6 AGCTCAGGGGGAGAATACCACTTCTCCCCtGGCGACATAATGTAAATGgtGCAGTTTGCCTCGAAATACTCCAGCTGCCCTGGAGTCATTTCCTTCATCC 1494

MSV-B AGCaCAGGGGGAGAATACCACTTCTCCCCtGGCGACATttTaTAAATcAtGCAGTTTGCCTCGAAATACTCCAtCTGCCCTGGAGTCATTTCCTTCATCC 1492

Consensus agc cagggggagaatacca ttctcccc ggcgacat t taaat gcagtttgcctcgaaata tcca ctgccctgga tcatttccttcatcc

MSV-A1 AATCTTCATCCGAGTTGGCGAGGATTATTGTAGGCTTAGACTTCTTCTGCACCTTCTTCTTCTTACCATACTTGGGGTTCACAATGAAATCCCTCTGACA 1596

MSV-A2 AATCTTCATCCGAGTTGGCGAGGATTATTGTAGGCTTAGACTTCTTCTGCACCTTtTTCTTtTTACCATACTTGGGGTTtACAATGAAATCCCTCTGACA 1594

MSV-A3 AATCTTCATCCGAGTTGGCGAGGATTATTGTAGGCTTAGACTTCTTCTGCACCTTCTTCTTCTTACCATAtTTGGGGTTtACAATGAAATCCCTCTGACA 1594

MSV-A4 AATCTTCATCCGAGTTGGCGAGGATTATTGTAGGCTTAGACTTCTTCTGCACCTTtTTCTTCTTACCATACTTGGGGTTtACAATGAAATCCCTCTGACA 1597

MSV-A6 AATCTTCATCCGAGTTGGCGAGGATTATTGTAGGCTTAGACTTCTTCTGCACCTTCTTCTTCTTACCATACTTGGGGTTgACAATGAAATCttTCTGgCA 1594

MSV-B AgTCTTCATCCcAGTTGGtGAGGATgATTGTAGGCTTAGACTTCaTCTGaACtTTCTTCTTCTTgCCATAtTTtGGaTTtACcAcGAAcTCttTCTGACA 1592

Consensus a tcttcatcc agttgg gaggat attgtaggcttagacttc tctg ac tt ttctt tt ccata tt gg tt ac a gaa tc tctg ca

Potential polyadenylation signal for small C-sense transcript

MSV-A1 GCCAACTAACTGTTTCCAACAAGGACAAAATTTAAACGGAATATCATCTACGATGTTGTAGATTGCGTCTTCGTTGTATGAAGACCAATCAACATTATTT 1696

MSV-A2 GCCAACTAACTGTTTCCAACAAGGACAgAATTTAAACGGAATATCATCTACGATGTTaTAGATTGCGTCTTCGTTGTATGAAGACCAATCAACATTATTT 1694

MSV-A3 GCCAACTAACTGTTTCCAACAAGGACAgAATTTAAACGGAATATCATCTACGATGTTGTAGATTGCGTCTTCGTTGTATGAAGACCAATCAACATTATTT 1694

MSV-A4 GCCAACTAACTGTTTCCAACAAGGACAgAATTTAAACGGAATATCATCTACGATGTTGTAGATTGCGTCTTCGTTGTATGAAGACCAATCAACATTATTT 1697

MSV-A6 GCCAACTAACTGTTTCCAACAAGGACAgAATTTAAACGGAATATCATCTACGATGTTGTAGATTGCGTCTTCGTTGTATGAAGACCAATCAACATTATTT 1694

MSV-B GCCAACTAACTGTTTCCAACAAGGACAgAATTTAAAaGGAATATCATCTACGATGTTGTAGATTGCGTCTTCGTTGTAcGAAGACCAATCAACATTATTT 1692

Consensus gccaactaactgtttccaacaaggaca aatttaaa ggaatatcatctacgatgtt tagattgcgtcttcgttgta gaagaccaatcaacattattt

RepA termination codon

MSV-A1 TGCCAGTAATTATGAACCCCTAGGCTTCTGGCCCAAGTAGATTTTCCGGTTCTTGTTGGGCCGACGATGTAGAGGCTCTGCTTTCTTGATCTTTCATCTG 1796

MSV-A2 TGCCAGTAATTATGAACCCCTAGGCTTCTGGCCCAAGTAGATTTTCCGGTTCTTGTTGGGCCGACGATGTAGAGGCTCTGCTTTCTTGATCTTTCATCTG 1794

MSV-A3 TGCCAGTAATTATGAACCCCTAGGCTTCTGGCCCAAGTAGATTTTCCGGTTCTTGTTGGGCCGACGATGTAGAGGCTCTGCTTTCTTGATCTTTCATCTG 1794

MSV-A4 TGCCAGTAATTATGAACCCCTAGGCTTCTGGCCCAAGTAGATTTTCCGGTTCTTGTTGGGCCGACGATGTAGAGGCTCTGCTTTCTTGATCTTTCATCTG 1797

MSV-A6 TGCCAGTAATTATGAACCCCTAGGCTTCTGGCCCAAGTAGAcTTTCCtGTTCTTGTTGGcCCGACGATGTAGAGGCTCTGCTTTCTTGATCcTTCATCTG 1794

MSV-B TGCCAaTAATTATGAAgCCCTAaGCTTCTGGCCCAAGTAGATTTTCCtGTTCgTGTTGGaCCGACGATGTAGAGGCTCTGCTTTCTTGtTCcTTCATCTG 1792

Consensus tgcca taattatgaa cccta gcttctggcccaagtaga tttcc gttc tgttgg ccgacgatgtagaggctctgctttcttg tc ttcatctg

Branch Point

Acceptor site

Intron

Donor site

MSV-A1 ATGACTGGATACAGAATCCATCCATTGGAGGTCAGAAATTGCATCCTCGAGGGTATAACAGGTAGGTTGAAGGAGCATGTAAGCTTCGGGACTAACCTGG 1896

MSV-A2 ATGACTGGATACAGAATCCATCCATTGGAGGTCAGAAATTGCATCCTCGAGGGTATAACAGGTAGGTTGAAGGAGCATGTAAGCTTCGGGACTAACCTGG 1894

MSV-A3 ATGACTGGATACAGAATCCATCCATTGGAGGTCAGAgATTGCATCCTCGAGtGTATAACAGtTAGGTTGAAGGAGCATGTAAGCTTCGGGACTAACCTGa 1894

MSV-A4 ATGACTGGATACAGAATCCATCCATTGGAGGTCAGAAATTGCATCCTCGAGGGTATAACAGGTAGGTTGAAGGAGCATGTAAGCTTCGGGACTAACCTGG 1897

MSV-A6 ATGACTGGATACAGAATCCATCCATTGGAGGTCAGAgATTGCATCCTCGAGGGTATAACAGGTAGGTTGAAGGAGCATGTAAGCTTCGGGACTgACCTGG 1894

MSV-B tTGgCTGGATAattgATCCATCCATTcaAGGTCAGAgATaGCcTCtTCGAGGGTgTAACAGGTtGGTTGAAGaAGCATGTAtGaTTCGGGACTAACCTGG 1892

Consensus tg ctggata atccatccatt aggtcaga at gc tc tcgag gt taacag t ggttgaag agcatgta g ttcgggact acctg

MSV-A1 AAGATGTTAGGCTGGAGCCAATCATTGATTGACTCATTACAAAGTAAATCAGGTGAGGAGGGTGGATGAGGATTGGTGAACTCTTCCTGAATCTCAGGAA 1996

MSV-A2 AAGATGTTAGGCTGGAGCCAATCATTGATTGACTCATTACAAAGTAAATCAGGTGAtGAGGGTGGATGAGGATTGGTGAACTCTTCCTGAATCTCAGGAA 1994

MSV-A3 AAGATGTTAGGCTGGAGCCAATCATTGATTGACTCATTACAgAGcAAATCAGGTtgGGAGGGTGGATGAGGATTGGTGAACTCTTCCTGAATCTCAGGAA 1994

MSV-A4 AAGATGTTAGGCTGGAGCCAATCgTTGATTGACTCATTACAAAGTAAATCAGGTGAGGAGGGTGGATGAGGATTGGTGAACTCTTCCTGAATCTCAGGAA 1997

MSV-A6 AAGATGTTAGGCTGGAGCCAATCATTGATTGACTCATTACAgAGTAAATCAGGTGAGGAGGGTGGATGAGGATTGGTGAACTCTTCCTGAATtTCAGGAA 1994

MSV-B AAGATGTTAGGCTGGAGCCAATCtTTGATTGACTCATTACAAAGaAgATCAGGTGAaGAGGGTGGATGAGGAcTGaTGAACTCTTCCTGAATCTCAGGgA 1992

Consensus aagatgttaggctggagccaatc ttgattgactcattaca ag a atcaggt gagggtggatgagga tg tgaactcttcctgaat tcagg a

MSV-A1 AAAGCTTATTTGCAGAGTATTCAAAATACTGCAATTTTGTGGACCAATCAAAGGGAAGCTCTTTCTGGATCATGGAGAGGTACTCTTCCTTGGAAGTAGC 2096

MSV-A2 AAAGCTTATTTGCAGAGTATTCAAAATACTGCAATTTTGTGGACCAATCAAAGGGAAGCTCTTTCTGGATCATGGAGAGGTACTCTTCtTTGGAAGTAGC 2094

MSV-A3 AAAGCTTATTTGCAGAGTATTCAAAATACTGCAATTTTGTGGACCAATCAAAGGGAAGCTCTTTCTGGATCATGGAGAGGTACTCTTCCTTGGAAGTAGa 2094

MSV-A4 AAAGCTTATTTGCAGAGTATTCAAAATACTGCAATTTTGTGGACCAATCAAAGGGgAGCTCTTTCTGGATCATGGAGAGGTACTCTTCtTTGGAgGTAGC 2097

MSV-A6 AAAGtTTATTTGCAGAGTATTCAAAATACTGCAATTTTGTGGACCAATCAtAGGGAAaCTCTTTCTGGATCATaGAGAGGTACTCTTCCTTGGAgGTgGa 2094

MSV-B AcAatTTATTTGCAGAGTATTCAAAATACTGCAATTTTGTGGcCCAgTCAtAGGGgAaCTCTTTacGaAcCATaGAGAGGTACTCTagCTTtGAAGTgGa 2092

Consensus a a ttatttgcagagtattcaaaatactgcaattttgtgg cca tca aggg a ctcttt g a cat gagaggtactct tt ga gt g

MSV-A1 GTGTGAAATAATGTCTCGCATTATTTCATCTTTGGAAGGTTTTTTTTC...CTTAACTTCTGAATCAGATTTTCCTAGGAAGGGGGACTTCCTAGGAATG 2193

MSV-A2 GTGTGAAATAATGTCTCGCATTATTTCATCTTTaGAAGGcTTTTTTTC...CTTtACcTCTGAATCAGATTTTCCTAGGAAGGGGGACTTCCTAGGAATG 2191

MSV-A3 GTGTGAAATAATGTCTCGCATTATTTCATCTTTGGAAGGcTTTTTTTC...CTTtACTTCTGAATCAGATTTTCCTAGGAAGGGGGACTTCCTAGGAATG 2191

MSV-A4 GTGTGAAATAATGTCTCGCATTATTTCATCTTTaGAAGGcTTTTTTTC...CTTtACcTCTGAATCAGATTTTCCTAGGAAGGGGGACTTCCTAGGAATG 2194

MSV-A6 GTGTGAAATAATGTCTCGCATTATTTCATCTTTaGAAGGTTTTTTTTC...CTTtAacTCTGAATCAGATTTTCCTAGGAAGGGGGACTTCCTAGGAATG 2191

MSV-B GTGTGAAATAATcTCTCtCATTATTTCATCTTTGGAAGGTTTcTTTTCagaaTTtcCcTtgGA...AGgaTTTCCTAGGAAGcttGACTTCCTAGGAATG 2189

Consensus gtgtgaaataat tctc cattatttcatcttt gaagg tt ttttc tt t ga ag tttcctaggaag gacttcctaggaatg

MSV-A1 AAAGTACCTCTCTCAAAAACAGCCAGAGGTTCCTTGAGAATGTAATCCCTCACCCTGTTTACTGATTTGGCACTCTGAATGTTTGGGTGAAACCCATTAA 2293

MSV-A2 AAAGTACCTCTCTCAAAcACAGCCAGAGGTTCCTTGAGAATGTAATCCCTCACCCTGTTTACTGAcTTGGCACTCTGAATaTTTGGGTGAAACCCATTtA 2291

MSV-A3 AAAGTACCTCTCTCAAAcACAGCCAGAGGTTCCTTGAGAATGTAATCCCTCACCCTGTTaACTGAcTTGGCACTCTGAATaTTTGGGTGAAACCCATTtA 2291

MSV-A4 AAAGTACCTCTCTCAAAcACAGCCAGAGGTTCCTTGAGAATGTAATCCCTCACtCTGTTaACTGAcTTGGCACTCTGAATaTTTGGGTGAAACCCATTtA 2294

MSV-A6 AAAGTACCTCTCTCAAAcAtAGCCAGAGGTTCCTTGAGAATGTAATCtCTCACCCTGTTgACTGAcTTGGCACTCTGAATaTTTGGGTGAAACCCATTtA 2291

MSV-B AAAGTACCTCTCTCAAAcAagGCCAGAGGTTCCTTGAGAATGTAATCCCTgACCtTGTTTACTGATTTtGCACTCTGAATGTTTGGGTGgAACCCtTTgA 2289

Consensus aaagtacctctctcaaa a gccagaggttccttgagaatgtaatc ct ac tgtt actga tt gcactctgaat tttgggtg aaccc tt a

MSV-A1 TATCAAAGAACCTTGAGTCGGATATCCTTACCGGCTTCTCTGTCTGAAGTAATGCATGTAAATGCAAACTTCCATCTTTATGTGCCTCTCGGGCACATAG 2393

MSV-A2 TATCAAAGAACCTTGAGTCaGATATCCTTACCGGCTTCTCTGTCTGAAGcAATGCATGTAAATGCAAACTTCCATCTTTATGTGCCTCTCGGGCACATAG 2391

MSV-A3 TATCAAAGAACCTTGAGTCaGATATCCTTACCGGCTTCTCTGTCTGAAGcAATGCATGTAAATGCAAACTTCCATCTTTATGTGCCTCTCGGGCACATAG 2391

MSV-A4 TATCAAAGAACCTTGAGTCaGATATCCTTAtCGGCTTCTCTGgCTGAAGcAATGCATGTAAATGCAAACTTCCATCTTTATGTGCCTCTCGGGCACATAG 2394

MSV-A6 TATCAAAGAACCTTGAGTCaGATATCCTTACCGGCTTCTCTGTCTGAAGcAATGCATGTAAATGCAAACTTCCATCTTTATGTGCCTCTCGGGCACATAG 2391

MSV-B TATCAAAGAACCTTGAGTCaGtgATtCTTACCGGtTTCTCTGTCTGgAtTAATGCATGTAAATGCAtAtcTCCATCTcTATGTGCCTCTCGGGCACATAt 2389

Consensus tatcaaagaaccttgagtc g at ctta cgg ttctctg ctg a aatgcatgtaaatgca a tccatct tatgtgcctctcgggcacata

MSV-A1 AATGTATTTGGGAATCCAACGAACAACGAGCTCCCAGATCATCTGACAGGCGATTTCAGGATTTTCTGGACACTTTGGATAGGTGAGGAACGTGTTAGCG 2493

MSV-A2 AATGTATTTGGGAATCCAACGAACAACGAGCTCCCAGATCATCTGACAGGCGATTTCAGGATTTTCTGGACACTTTGGATAGGTtAGGAACGTGTTAGCG 2491

MSV-A3 AATaTATTTGGGAATCCAACGAACAACGAGCTCCCAGATCATCTGACAGGCGATTTCAGGATTTTCTGGACACTTTGGgTAcGTtAGGAACGTGTTAGCG 2491

MSV-A4 AATaTATTTGGGAATCCAACGAACgACGAGCTCCCAGATCATCTGACAGGCGATTTCAGGATTTTCTGGACACTTTGGATAGGTtAGGAACGTGTTAGCG 2494

MSV-A6 AATaTAcTTGGGtATCCAACGAACAACGAGCTCCCAaATCATCTGACAGGCGATTTCAGGATTTTCTGGACACTTTGGATAGGTaAGGAACGTGTTAGCG 2491

MSV-B gATaTAcTTcGGggTCCAACGAcCAACGAGCTCCCAaATCATCTGACAGagGATTTCtGGATTTTCTGGACAgTgTGGATAtGTtAaGAACGTGTTAaCG 2489

Consensus at ta tt gg tccaacga c acgagctccca atcatctgacag gatttc ggattttctggaca t tgg ta gt a gaacgtgtta cg

RepA gene

TATA box

*RepA* start codon

MSV-A1 TTCCTGTGTGAGAACTGACGGTTGGATGAGGAGGAGGCCATAGCCGACGACGGAGGATGAGGCTGAGGGATGGCAGACTGGGAGCTCCAAACTCTATAGT 2593

MSV-A2 TTCCgGTGTGAGAACTGACGGTTGGATGAGGAGGAGGCCATtGCCGACGACGGAGGtTGAGGCTGAGGGATGGCAGACTGGGAGCTCCAAACTCTATAGT 2591

MSV-A3 TTCCTGTGTGAGAACTGACGGTTGGATGAGGAGGAGGCCATAGCCGACGACGGAGGcTGAGGCTGAGGGATGGCAGAaTGGGAGCTCCAAACTCTATAGT 2591

MSV-A4 TTCCTGTGTGAGAACTGACGGTTGGATGAGGAGGAGGCCATAGCCGACGACGGAGGtTGAGGCTGAGGGATGGCAGACTGGGAGCTCCAAACTCTATAGT 2594

MSV-A6 TTCCTGTGTGAGAACTGACGGTTGGATGAGGAGGAGGCCATgaCCGACGACGGAGGAcGtGGCTGAGcGATGGCAGAtTGGGAGCTCtcAACTCTATAac 2591

MSV-B TTCCTGTGTGAGAACTGACGGTTGGATGAGGAGGAGGCCATAtCgGACGACtccGagc.AGctTGAGGGATGGCAGgaTGGGAGCTCCAAACTCTATAtc 2588

Consensus ttcc gtgtgagaactgacggttggatgaggaggaggccat c gacgac g g tgag gatggcag tgggagctc aactctata

Stem Sequence

GC- box

Sequences repeated in the stem

MSV-A1 ATACCCGTGCGCCTTCGAAATCCGCCGCTCCCTTGTCTTATAGTGGTTGTAAATGGGCCGGACCGGGCCGGCCCAGCAGG.AAAAGAAGGCGCGCAC 2689

MSV-A2 ATACCCGTGCGCCTTCGAAATCCGCCGCTCCCTTGTCTTATAGTGGTTGcAAATGGGCCGGACCGGGCCGGCCCAGCAGG.AAAAGAAGGCGCGCAC 2687

MSV-A3 ATACCtGTGCGCCTTCGAAATCCGCCGCTCCCTTGTCTTATAGTGGTTGTAAATGGGCCGGACCGGGCCGGCCCAGCAGG.AAAAGAAGGCGCGCAC 2687

MSV-A4 ATACCCGTGCGCCTTCGAAATCCGCCGCTCCaTTGTCTTATAGTGGTTGTAAATGGGCCGGACCGGGCCGGCCCAGCAGG.AAAAGAAGGCGCGCAC 2690

MSV-A6 A.ACCgGTGCGCCTTCGAAATCCGCCGCTCC.TcGTtTTATAGTGGTTGTgtATGGGCCGGACCGGGCCGGCCCAGCAGG.AAAAGAAGGCGCGCAC 2685

MSV-B AacCggtTGCGCCTTCGAAATCCGCCGCTCCCcc.TtTTATAGTGGTTGTttATGGGCCGGACCGGGCCGGCCCAGCAGG.AAAAGAAGGCGCGCAa 2683

Consensus a c tgcgccttcgaaatccgccgctcc t ttatagtggttg atgggccggaccgggccggcccagcagg aaaagaaggcgcgca

- Under the line indicating the ‘consensus’ sequence;

Light blue rectangles indicate sites under negative selection in MSV (detected using the SLAC, REL, or IFEL methods for detecting selection).

Red rectangles indicate sites under positive selection in MSV (detected using the SLAC, REL, or IFEL methods for detecting selection).

- The mutations detected in the recombinant MSV, as well as wild type MSV-MatA and MSV-VW are indicated within the ‘comment’ boxes (see also Additional file 6).
