# Supplementary material for: Adaptive evolution by recombination is not associated with increased mutation rates in Maize streak virus
Source: BMC Evol Biol. 2012 Dec 27;12:252. doi: 10.1186/1471-2148-12-252 (PMC3556111; doi:10.1186/1471-2148-12-252)
Supplement: Additional file 11 — Nucleotide mutation dynamics based on the observed number mutations occurring between pairs of nucleotides during short-term evolution studies using MSV and TYLCV. The relative proportion of each mutation is expressed in each cell as the percentage of the total number of observed mutations under each experimental condition (the most commonly observed mutations are shown in bold). Cells in grey indicate instances in which no statistically significant (i.e. p < 0.05) difference was obtained between the observed and expected number of mutations; those in blue and red, however, indicate respectively instances where significantly less than expected, or significantly more than expected mutations were observed. The file is in .pdf format. [file 1471-2148-12-252-S11.pdf]

To

VWMPCPLIRMat

From	A	G	C	T
A		6.3	12.5	6.3
G	6.3		0	18.8
C	6.3	12.5		**18.8**
T	0	6.3	6.3	

MatMPCPVW+VWMPCPMat

From	A	G	C	T
A		7.5	7.5	3.5
G	7.5		3.7	**25.9**
C	3.7	3.7		22.2
T	11.1	0	3.7	

MatMPCPLIRVW+VWMPCPLIRMat

From	A	G	C	T
A		0	0	16.7
G	16.7		0	8.3
C	8.3	0		**41.7**
T	0	0	8.3	

MSV-MatA

From	A	G	C	T
A		15.4	11.5	7.7
G	11.5		3.8	3.8
C	7.7	7.7		**23.1**
T	3.8	3.8	0	

MSV-VW

From	A	G	C	T
A		3.4	0	3.4
G	17.2		3.4	3.4
C	6.9	3.4		**20.7**
T	17.2	17.2	3.4	

Combined MSV data

From	A	G	C	T
A		8.2	5.5	4.5
G	11.8		2.7	11.8
C	6.4	5.5		**23.6**
T	8.2	6.4	3.6	

TYLCV

From	A	G	C	T
A		2.7	3.4	5.1
G	16.4		4.5	15.8
C	10.3	5.8		**18.8**
T	5.5	4.1	7.5	

Additional file 11. Nucleotide mutation dynamics based on the observed number mutations occurring between pairs of nucleotides during short-term evolution studies using MSV and TYLCV. The relative proportion of each mutation is expressed in each cell as the percentage of the total number of observed mutations under each experimental condition (the most commonly observed mutations are shown in bold). Cells in grey indicate instances in which no statistically significant (i.e. $p < 0.05$) difference was obtained between the observed and expected number of mutations; those in blue and red, however, indicate respectively instances where significantly less than expected, or significantly more than expected mutations were observed.
